# Regulation of Early Steps of GPVI Signal Transduction by Phosphatases: A Systems Biology Approach

**DOI:** 10.1371/journal.pcbi.1004589

**Published:** 2015-11-19

**Authors:** Joanne L. Dunster, Francoise Mazet, Michael J. Fry, Jonathan M. Gibbins, Marcus J. Tindall

**Affiliations:** 1 Department of Mathematics and Statistics, University of Reading, Reading, Berkshire, United Kingdom; 2 Institute for Cardiovascular and Metabolic Research and School of Biological Sciences, University of Reading, Reading, Berkshire, United Kingdom; Northeastern University, UNITED STATES

## Abstract

We present a data-driven mathematical model of a key initiating step in platelet activation, a central process in the prevention of bleeding following Injury. In vascular disease, this process is activated inappropriately and causes thrombosis, heart attacks and stroke. The collagen receptor GPVI is the primary trigger for platelet activation at sites of injury. Understanding the complex molecular mechanisms initiated by this receptor is important for development of more effective antithrombotic medicines. In this work we developed a series of nonlinear ordinary differential equation models that are direct representations of biological hypotheses surrounding the initial steps in GPVI-stimulated signal transduction. At each stage model simulations were compared to our own quantitative, high-temporal experimental data that guides further experimental design, data collection and model refinement. Much is known about the linear forward reactions within platelet signalling pathways but knowledge of the roles of putative reverse reactions are poorly understood. An initial model, that includes a simple constitutively active phosphatase, was unable to explain experimental data. Model revisions, incorporating a complex pathway of interactions (and specifically the phosphatase TULA-2), provided a good description of the experimental data both based on observations of phosphorylation in samples from one donor and in those of a wider population. Our model was used to investigate the levels of proteins involved in regulating the pathway and the effect of low GPVI levels that have been associated with disease. Results indicate a clear separation in healthy and GPVI deficient states in respect of the signalling cascade dynamics associated with Syk tyrosine phosphorylation and activation. Our approach reveals the central importance of this negative feedback pathway that results in the temporal regulation of a specific class of protein tyrosine phosphatases in controlling the rate, and therefore extent, of GPVI-stimulated platelet activation.

## Author Summary

Platelets are blood cells that, upon injury, trigger the blood to clot. Following blood vessel damage platelets encounter the extracellular matrix protein collagen to which they respond. They become activated, aggregating to form a major component of blood clots. The platelet collagen receptor GPVI stimulates platelet activation through a complex signalling pathway, and while many of the molecules involved in the activation of this pathway have been identified, their specific roles in determining the rate and extent of the exceptionally rapid platelet response have not been determined. Furthermore, while signalling proteins responsible for forward reactions are known, reverse or negative feedback elements are not well understood. Platelets also trigger thrombosis in diseased arteries, causing heart attacks and strokes, and therefore platelets, and particularly the GPVI signalling pathway, are therapeutic targets. To begin to understand the components in the GPVI signalling pathways that may represent tractable therapeutic targets we have developed a mathematical model of the key initiating events that occur upon stimulation of GPVI. In so doing, we have established the importance of a specific phosphatase-controlled negative feedback in determining the rate of initiation of platelet activation.

## Introduction

Platelets, small anuclear cells, are quiescent in undamaged blood vessels. They respond to injury by activating, triggering blood to clot. Whilst platelet activation is essential to prevent excessive bleeding at sites of injury, their inappropriate activation, for example as a consequence of vascular disease, can lead to the formation of clots within the circulation, or thrombosis which triggers heart attacks and strokes [1].

Damage to blood vessels results in the exposure of extracellular matrix proteins, particularly collagens, which form structural components within the vessel wall. Collagens provide an initiating signal for platelet activation. Indirect interactions between platelets and collagen, in the arterial circulation, are mediated by von Willebrand factor (vWF) which binds to collagen and to GPIb on the platelet's surface. This causes platelets to slow down and roll along the site of vessel damage allowing direct binding to platelet collagen receptors, including integrin α2β1, which largely supports adhesion, and glycoprotein (GP) VI which stimulates cell signalling and activation [2]. Platelet activation is marked by a dramatic change in platelet shape, the secretion of various prothrombotic factors and conformational change in the integrin αIIbβ3. These secreted factors initiate a second wave of signalling. Integrin αIIbβ3 binding to fibrinogen, which in itself stimulates signalling, supports platelet aggregate formation allowing the assembly of a platelet thrombus (or haemostatic plug) to stem the loss of blood [2,3].

**Fig 1:**
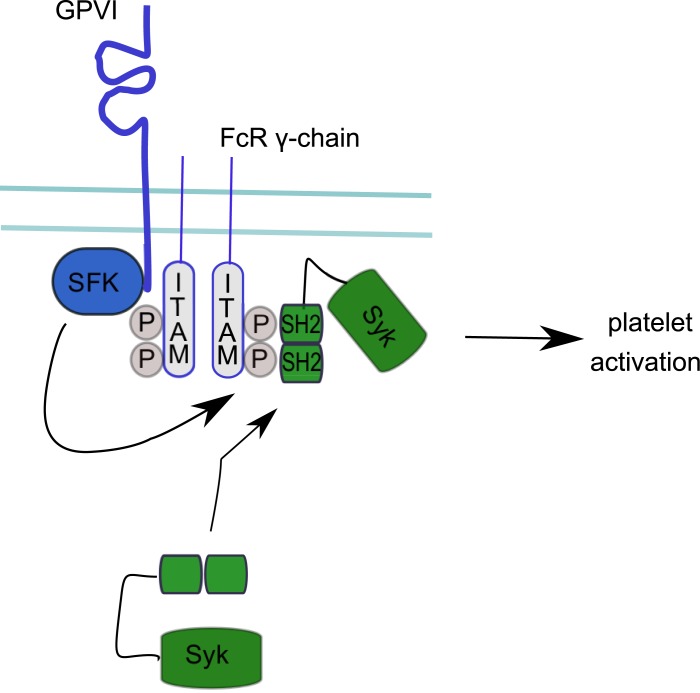
A schematic representation of GPVI signal transduction in a platelet. GPVI is present as a complex associated with Fc receptor (FcR) γ-chain and Src family kinases (SFK) that phosphorylate an immunoreceptor tyrosine based activation motif (ITAM) in the FcR γ-chain. This allows recruitment and activation of the cytosolic tyrosine kinase Syk that leads to phosphorylation of adaptor proteins such as LAT (linker for Activation of T-cells), PI3K (phosphoinositide 3-kinase) and ultimately activation of PLCγ2 (phospholipase C). These molecules participate in triggering the downstream processes of calcium release, shape change and granule secretion that all form part of platelet activation. Arrows are not intended to imply direct phosphorylation or activation.

GPVI is a member of the immunoglobulin family of receptors that shares aspects of its mechanisms of activation with immunoreceptors, including B and T cell antigen receptors [4]. These receptors possess or associate with transmembrane proteins that have cytoplasmic domains that contain immunoreceptor tyrosine-based activation motifs (ITAMs). The binding of a ligand leads to associated Src Family Kinases (SFKs) phosphorylating conserved tyrosine residues on the ITAM. In the case of GPVI, Syk (spleen tyrosine kinase), or the related protein ZAP70 in T cells, is recruited to the receptor complex through direct binding to the phosphorylated ITAM. Recruitment of Syk in turn provokes its phosphorylation [5], pivotal to downstream signalling events: in platelets this leads to activation, shape change and aggregation (Fig 1) [6]. The development of new drugs to suppress platelet function and thereby prevent thrombosis has been shown to be an effective strategy [7–9]; however, current anti-platelet therapies are ineffective in many patients and are associated with side effects. A more detailed understanding of the signalling pathways that lead to platelet activation is needed to develop more sophisticated, effective and safer anti-platelet therapies [10]. GPVI is a candidate anti-platelet drug target [11,12] and the central role of Syk in immune cell signalling also makes it an attractive therapeutic option, with implications for the treatment of allergy, auto-immune diseases and haematological malignancies [13].

In other cell types mathematical and computational models have aided in the understanding of signalling pathways and assisted further experimental work [14–16]. The formation of blood clots and thrombi has long been of mathematical interest but, probably due to the ready availability of data, mathematical modelling has focussed on the coagulation cascade [17–23], i.e. the network of interactions between blood proteins that results in the formation of fibrin polymers that stabilise a clot. These models show promise in translating an individuals' coagulant profile into a measure of thrombotic risk [24,25]. Even though platelets are an ideal cell type to model (they lack a nucleus thereby removing the complexities of gene transcription and translation) it is only recently that the platelet has become of mathematical interest. The main focus of recent developments have been the interactions between a platelet and the coagulation cascade [26,27] and the role of platelets in a thrombus shaped by blood flow [28–31], there being several excellent reviews [32–34]. Where platelets have been studied in more detail investigations focus on the functional response of platelets to agonists [35] and the mechanics of signalling pathways [36–39]. Dandekar and co-workers [36,37] focused on platelet signalling mechanisms and the effects of prostacyclin, a molecule secreted from endothelial cells that contributes to platelets remaining in a quiescent state in the undamaged circulation. Their studies were based on platelet-specific data and simulated and tested different pharmacological conditions and the contributions of various platelet receptors. A detailed mathematical model [38] that captures current knowledge of platelet signal transduction through the ADP receptor is able accurately to predict calcium flux and ADP dose-response. This work was used as a basis for a model of PAR-1 mediated platelet activation [39] that includes the critical step of linking inside-out to outside-in integrin signalling. These latter two studies focus on the events downstream of G-protein coupled receptors through to intracellular calcium mobilisation, a critical step in platelet activation that is common to all activation pathways.

While GPVI activation is critical in health and disease no mathematical study has focussed on the platelet's response to collagen. The pathway of reactions downstream of the GPVI receptor is considerably more complex than those stimulated by G-protein coupled receptors. We therefore focus our investigations on the early reactions, proximal to the GPVI receptor, that lead to Syk activity that is thought to be a fundamental switch that controls GPVI mediated signal transduction.

Studies to dissect the component parts and associated processes within the GPVI signalling pathway have provided a picture of the series of steps that occur [40] but these studies often lack detailed kinetic analysis and molecular detail. For example, they may measure the level of tyrosine phosphorylation of a specific protein, rather than assessing the phosphorylation of specific amino acid residues, despite these individual phosphorylated residues being linked to specific functions. Furthermore, previous attempts to model signalling pathways in this and other cell types have often been based on qualitative data, which may make it difficult to detect differences between experimental data and model predictions. Here, these limitations in available data were resolved by the collection of high density quantitative experimental observations that capture the temporal increase and decrease in tyrosine phosphorylation of key proteins at specific amino acid residues in the early steps of activation of the GPVI signalling pathway.

Existing knowledge of platelet signalling pathways tends to focus on the sequential forward reactions that lead to platelet activation. While tight negative regulation of the pathways appears crucial for appropriate and rapid platelet activation, little effort has focussed on understanding what the key regulators are that modulate these reactions and how they integrate to precisely control platelet activation. Stimulation of GPVI results in the initiation of a signalling pathway that is highly dependent on the activities of a range of protein kinases. A protein tyrosine kinase transfers a phosphate group to a protein. Such phosphorylation may alter the function of a given protein, for example through inducing a conformational change, creating docking sites, causing intracellular relocation, and modulation of enzymatic activity. Protein tyrosine phosphatases reverse these protein modifications since they are able to remove phosphate groups returning proteins to their original state. Historically protein tyrosine kinases have been the focus of more research than protein tyrosine phosphatases. Recent studies indicate that human platelets possess at least 18 different protein tyrosine phosphatases, numbering more than 52,000 copies in total per platelet [41]. While traditionally phosphatases have been thought to be promiscuous in activity, constitutively active and able to remove phosphate moieties from proteins, it is now evident that this view is too simplistic and that phosphatases like kinases are actively recruited and regulated within specific pathways [10,42,43]. The identities and roles of phosphatases within the GPVI signalling pathway are not well understood, although it is clear that some play a fundamental role in the control of platelet function following stimulation with collagen [44,45,46].

Biological knowledge of components and interactions of signalling pathways is often expressed by cartoons or network diagrams. These descriptive static models provide little information about how the many components evolve over time, making it difficult to predict system behaviour. Here we reinterpret network diagrams, representing current biological knowledge and hypotheses surrounding the early regulation of the GPVI signalling pathway, into a series of dynamic mathematical models that include various possible modes of regulation by phosphatases. This allows direct comparison between knowledge and hypotheses and high density quantitative, temporal experimental data generated specifically to inform the development of the models. Through a process of parameter estimation, model validation and comparison with experimental data we gain understanding of the temporal dynamics that control the initiation of the GPVI signalling pathway allowing us to explore how perturbations of different kinds (e.g. ligand availability, negative regulation processes, and expression of key regulatory proteins) effect the ability of GPVI to signal downstream.

## Results

**Fig 2:**
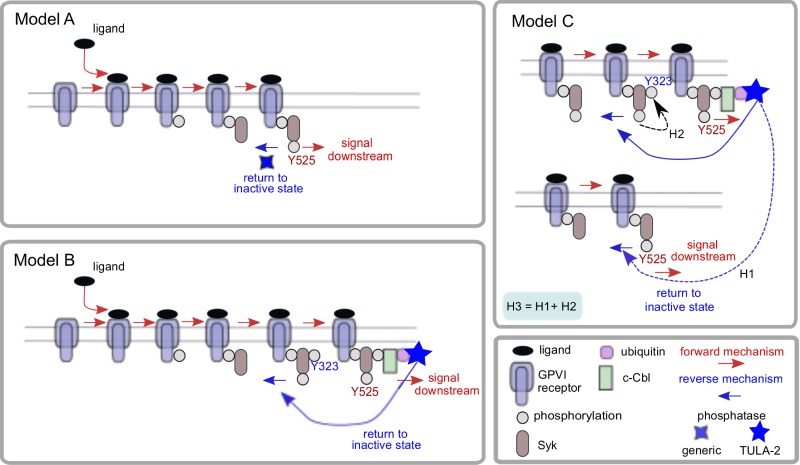
A schematic diagram of the reactions captured in each of our mathematical models. In all models GPVI, its associated Fc receptor γ-chain and Src family kinase, are treated as one unit. A ligand binds to this receptor bundle, which is subsequently phosphorylated, allowing for recruitment and activation of the cytosolic tyrosine kinase Syk. Binding to the receptor leads to auto-phosphorylation of Syk on tyrosine 525 (Y525), allowing the receptor to signal downstream. Model A incorporates a simple phosphatase that is able to dephosphorylate Syk on Y525, returning the receptor complex to an inactive state. In Model B an additional Syk phosphorylation site is incorporated (Y323), to which the newly introduced protein c-Cbl can bind. This allows Syk to set off a series of reactions, that include ubiquitination and the binding of the phosphatase TULA-2 that is able to dephosphorylate Syk on Y525, returning the receptor complex to an inactive state. In Model C two modifications are incorporated: the modification H1 allows TULA-2 to dephosphorylate not only the Syk molecule to which it is bound but also any nearby bound Syk molecule; in the second modification (H2) phosphorylation of Y525 results in an enhanced rate of Y323 phosphorylation. Model C, H3 incorporates H1 and H2 simultaneously.

**Fig 3:**
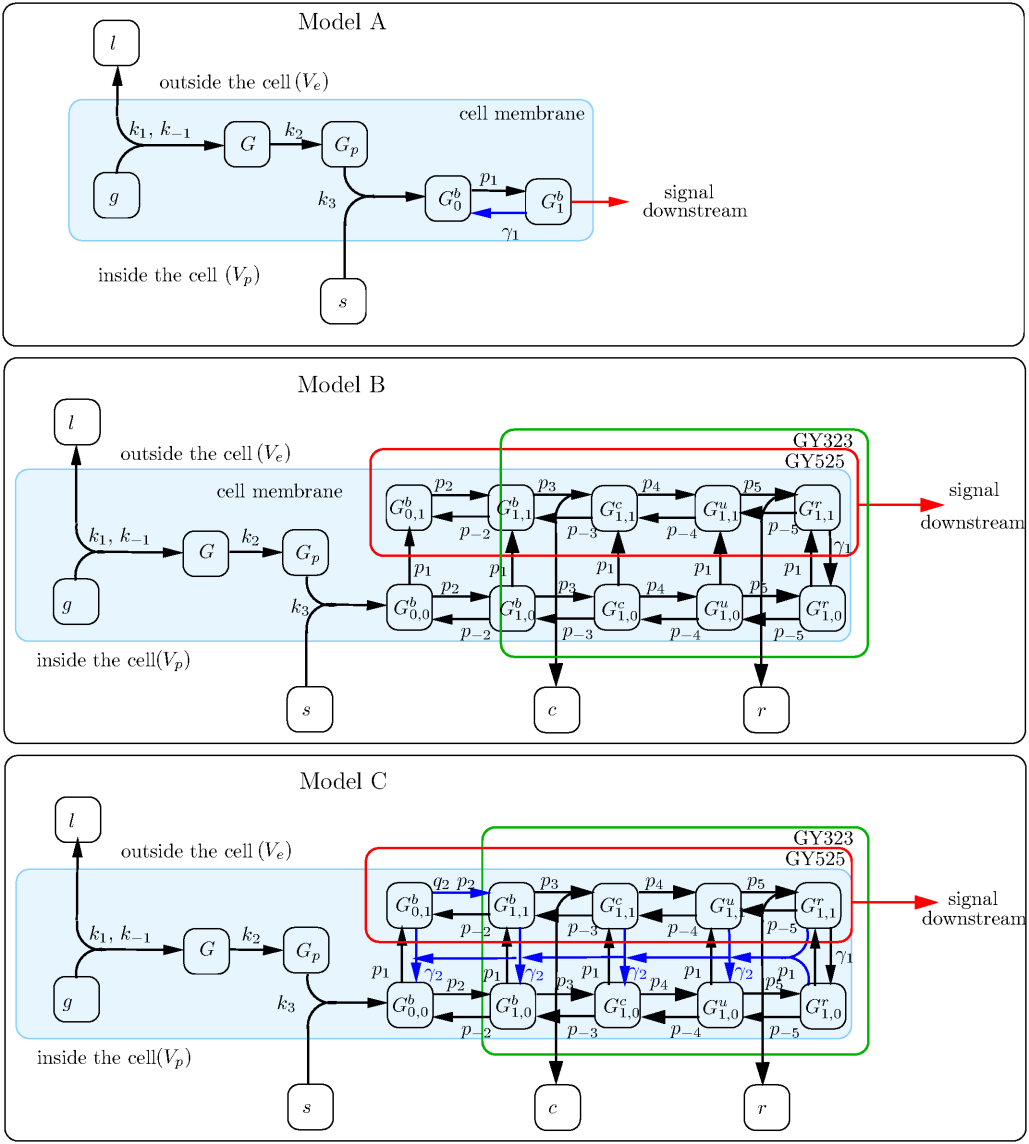
A network diagram of Model A (top panel), B (middle panel) and C (lower panels). Variables are represented by square boxes and the parameter associated with each processes is placed next to the relevant arrow. In all panels, from left to right, a ligand (*l*) binds to a free receptor (*g*) on the cell surface forming a receptor complex (*G*) that phosphorylates (*G*_*p*_). In Model A Syk (*s*) binds to the receptor complex ($G_{\text{0}}^{b}$) and activates ($G_{\text{1}}^{b}$). The active receptor can then signal downstream. In the middle and lower panels, the components encased in the red box represent Syk phosphorylation on Y525 (equation S6) and those in the green box represent Syk phosphorylation on Y323 (equation S7). See Tables 1 and 2 for a description of the variables and parameters.

We have developed a series of nonlinear ordinary differential equation (ODE) mathematical models to understand the early steps in signal transduction initiated by the GPVI receptor and how these might be regulated by phosphatases. Each model incorporates the same forward reactions that occur early in GPVI signal transduction and lead to the recruitment and phosphorylation of the cytosolic protein Syk (tyrosine 525 (Y525), used as a surrogate marker for Syk activity, and the ability of the receptor to signal downstream [47]). The mathematical models vary in their methods and details of regulation of Syk activity and the role of specific types of phosphatases. We assume that: the total surface area of a platelet, the density of its receptors and the concentration of cytosolic proteins remain constant over the time scale of interest; spatial variation is assumed not to be relevant and GPVI receptors and cytosolic proteins are evenly distributed over the surface of the platelet and throughout the cell cytosol, respectively. These assumptions allow us to translate the reactions incorporated in each model (using mass action kinetics) into systems of ordinary differential equations. Fig 2 provides a schematic diagram describing the reactions incorporated into each model and Fig 3 provides a network diagram of these events and the refinements made through models A to C, described in the following sections. Full details of the model equations are provided in S1 Text and a summary of model variables and parameters is presented in Tables 1 and 2 respectively.

**Table 1:** Model variables. The variables described in the top five rows are common to all models while the following two rows describe variables unique to Model A and the remaining rows describe variables unique to Model B and C. $G_{i,j}^{k}$ represents eight variables where i and j indicate phosphorylation on Y323 and Y525 respectively (0, unphosphorylated; 1, phosphorylated) and k denotes the sequential processes of (b), binding of Syk; (c), binding of c-Cbl; (u), ubiquitination and (r), binding of TULA-2.

Variable	Description	Units	Initial condition	Source
*l*	Ligand	moles m^-3^	3x10-2	estimated
*g*	GPVI	molecules	5000	this study
*s*	Syk, cytosolic	molecules	2763	this study
*G*	Ligand bound to GPVI	molecules	0	
*Gp*	Phosphorylated receptor	molecules	0	
$G_{\text{0}}^{b}$	Syk bound to receptor	molecules	0	
$G_{\text{1}}^{b}$	Syk phosphorylated on Y525	molecules	0	
$G_{i,j}^{k}$	Syk bound to receptor	molecules	0	
*r*	TULA-2	molecules	7800	[41]
*c*	c-Cbl	molecules	2581	this study

**Table 2:** A summary of model parameters. Their definitions, units, and values utilised in simulations. N/A indicates that the parameter plays no role in the specified model. Further details of the parameter values, their sources in literature and how they were obtained from the parameter fitting process are available in S1 Text.

Parameter	Description	Units	Values			Source
			Model A	Model B	Model C	
*k*_1_	rate of ligand binding	*m* ^3^moles^-1^ *s* ^-1^	8	8	8	literature
*k*_-1_	ligand dissociation constant	*s* ^-1^	3.02×10^−2^	3.02×10^−2^	3.02×10^−2^	literature
*V*_*e*_	extracellular volume per cell	*m* ^3^	3.3×10^−9^	3.3×10^−9^	3.3×10^−9^	this study
*V*_*p*_	platelet volume	*m* ^3^	7.4×10^−18^	7.4×10^−18^	7.4×10^−18^	this study
*K*_2_	rate of receptor phosphorylation	*s* ^-1^	3.02×10^−2^	4.57×10^−2^	4.71×10^−2^	fitting process
*K*_3_	rate Syk binds to receptor	*s* ^-1^	9.55×10^5^	9.55×10^5^	6.82×10^4^	fitting process
*p*_1_	rate Syk phosphorylates	*s* ^-1^	5.13×10^−1^	1.83×10^−1^	1.44×10^−2^	fitting process
*γ*_1_	rate Syk dephosphorylates	*s* ^-1^	3.53	9.96×10^2^	1.00×10^−2^	fitting process
*P*_2_	Syk phosphorylation (Y323)	*s* ^-1^	N/A	2.62×10^1^	4.48	fitting process
*p*_-2_	Syk Y323 regulation	*s* ^-1^	N/A	2.28×10^2^	2.16×10^1^	fitting process
*p*_3_	C-Cbl binding	*m* ^3^moles^-1^ *s* ^-1^	N/A	3.39×10^4^	5.67×10^2^	fitting process
*p*_-3_	c-Cbl dissociation	*s* ^-1^	N/A	7.24×10^1^	9.91	fitting process
*p*_4_	ubiquitination	*s* ^-1^	N/A	9.96×10^2^	1.73×10^−2^	fitting process
*p*_-4_	deubiquitination	*s* ^-1^	N/A	8.74×10^1^	3.02×10^−1^	fitting process
*p*_5_	TULA-2 binding	*m* ^3^moles^-1^ *s* ^-1^	N/A	2.11×10^4^	1.00×10^1^	fitting process
*p*_-5_	TULA-2 dissociation	*s* ^-1^	N/A	4.25×10^2^	4.94×10^−2^	fitting process
*γ*_2_	Dephosphorylation of Y525	molecules^-1^ *s* ^-1^	N/A	N/A	2.50×10^−1^	fitting process
*q*_2_	Increase in Syk Y323 phosphorylation	dimensionless	N/A	N/A	4.19×10^2^	fitting process

Changes in Syk phosphorylation at different amino acid residues over time were measured in platelets, obtained from three separate blood samples taken from one healthy donor. Isolated human platelets were stimulated using the multivalent GPVI-selective agonist collagen-related peptide (CRP) [5]. Secondary signalling events that may arise from the secretion or release of factors from stimulated platelets and could therefore interfere with events proximal to the GPVI receptor (e.g. ADP, thromboxane A_2_, integrin αIIbβ3 engagement), were suppressed using pharmacological agents. Site-specific protein phosphorylation was measured at twenty-two time points over a two hundred and fifty second interval, following stimulation. The results were quantified against reference standard curves compiled using recombinant protein and specific antibodies that recognise total Syk or Syk phosphorylated on specific tyrosine residues (see Methods for details describing how experimental data were collected and quantified). Each mathematical model was solved and compared with the experimental data. The discrepancies between model solutions and experimental observations were investigated, and, where necessary, the model structure was adapted. To solve the mathematical model numerically required values to be assigned to the initial conditions (protein copy numbers) and parameters (rates at which the proteins interact) that are captured in the models. Where possible total protein copy numbers were obtained by quantitative immunoblotting and parameters were estimated from our experimental data via a process of parameter fitting (see Methods for a description of the parameter fitting process) within boundaries indicated within the literature. Later, once validated, model profiles were compared to experimental observations describing Syk activity within a wider population of donors and under the effects of ligand depletion.

An issue in developing mathematical models to describe (often noisy) experimental data is the introduction of too much complexity (additional parameters) to ensure that the model solution fits available data (overfitted). To overcome this limitation, a widely applied information theory based approach (Akaike's information criterion (AIC)) was used to establish a ranking between competing models (see Methods). This metric utilises the results from the parameter fitting process, and characterise the trade off between goodness of fit and model complexity, allowing selection of the model with the smallest number of parameters, which still describes the data sufficiently well.

### Model A

In all models we treated GPVI, its associated Fc receptor γ-chain and SFKs as a single entity (see Fig 2). The ligand (CRP and indeed collagen) is a complex one being of indeterminant length, weight and number of binding sites for the GPVI receptor. While ligand mediated clustering is thought to be an important attribute the difficulty with obtaining experimental data covering these very early timepoints leads us to neglect these complications and assume an abundant ligand source. A ligand binds to this receptor bundle, which is subsequently phosphorylated, allowing for recruitment and activation of the cytosolic protein-tyrosine kinase Syk. Binding to the receptor leads to auto-phosphorylation of Syk, on tyrosine 525 (Y525), that allows the receptor to signal downstream [48–50]. The early reactions of ITAM phosphorylation and Syk binding are unlikely to be rapidly reversible. The binding affinity of Syk to the ITAM is high, occurring through two tyrosine residues [5] that protect the ITAM from dephosphorylation. Experimental data shows quantitative differences between protein copy numbers and equilibrium levels of Syk phosphorylation, pointing to regulatory activity. Syk phosphorylation is an obvious candidate for regulation, most likely through a phosphatase. Phosphatases have traditionally been viewed as promiscuous housekeeping molecules that act quickly on exposed phosphoproteins [43]. Our initial model (A) incorporated the continuous presence of a simple abundant generic phosphatase that is able to dephosphorylate Syk on Y525, thereby restricting the ability of Syk (and therefore of the GPVI receptor) to signal downstream.

**Fig 4:**
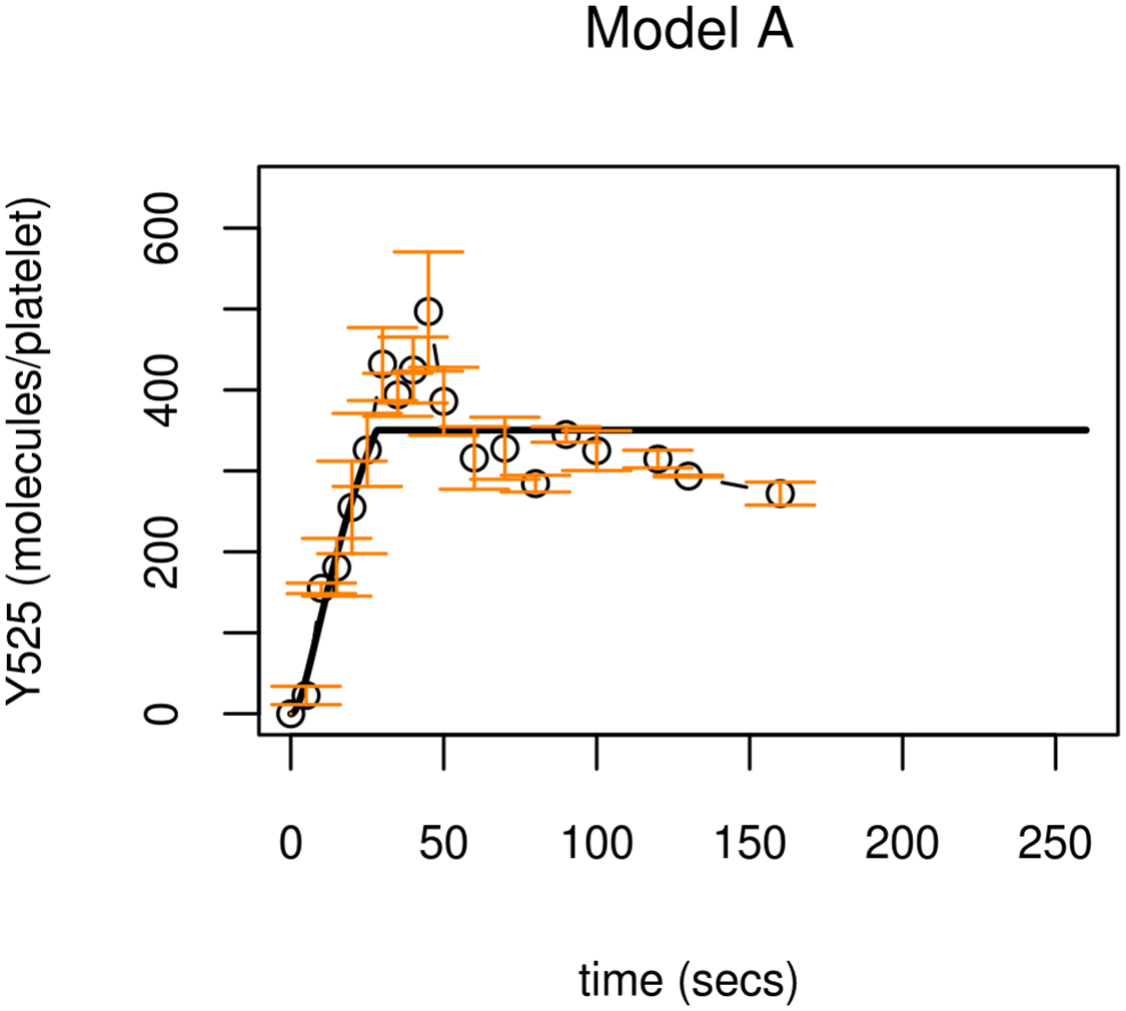
Model A profiles compared to experimental observations of Syk phosphorylation on Y525. Model profiles (black lines) compared to experimental observations (circles). The model accurately describes the steady state seen in experimental observations but is unable to describe the early peak in Syk Y525 phosphorylation. Experimental data of Syk phosphorylation (molecules per platelet) is the mean of three samples +/- S.E.M., phosphorylated (circles). Model simulations (solid lines) utilise parameter values from Table C in S1 Text (parameter set 1).

Full details of the model and the parameter values obtained are given in S1 Text. Model solutions describing Syk phosphorylation monotonically rise and settle to the steady state seen in experimental data (see Fig 4, top row). The steady state is solely determined by Syk's rate of phosphorylation and its regulation, such that

$$\text{Bound, inactive Syk }=\frac{\gamma_{\text{1}}}{p_{\text{1}}+\gamma_{1}}s_{I}\text{, Active Syk }=\frac{p_{\text{1}}}{\gamma_{\text{1}}+p_{1}}s_{I},$$ (1)

Where *s*_*I*_ denotes Syk copy numbers, *p*_1_ the rate that Syk is phosphorylated and *γ*_1_ the rate at which it is dephosphorylated. Local sensitivity analysis (see Methods) reveals that the model's steady state is insensitive to variation in the rate of ITAM phosphorylation and the rate that Syk binds to the phosphorylated ITAM.

While Model A is able to describe the equilibrium to which Syk activity settles, it is unable to capture its early transient peak in phosphorylation. This early peak in Syk phosphorylation is reminiscent of negative regulatory behaviour affecting Syk activity and points to the constitutively activated phosphatase being insufficient to explain this behaviour. Therefore the model was adapted, replacing the 'housekeeping' phosphatase with a more complex pathway that involves a specific phosphatase, TULA-2. While it is known that TULA-2 can interact with Syk, in a ubiquitin-dependent manner [51], the purpose of the interaction had not previously been established. This interaction becomes the template for model adaption.

### Model B

The mathematical model was extended to incorporate a negative feedback, which leads from Syk phosphorylation to its own regulation, with the intention of explaining the early peak in Syk activity seen in experimental data. This newly introduced regulatory pathway centres on the proteins c-Cbl and TULA-2. c-Cbl is a member of the Cbl (Casitas B-lineage lymphoma) family of adaptor proteins and is known to be found in platelets [50,52]. It has been shown to play a role in regulating signals by similar receptors in other cell types [53,54] and it has been implicated in GPVI regulation in platelets [52]. c-Cbl is a ubiquitin ligase that is able to associate with phosphorylated tyrosine kinases, such as Syk, whereby it can promote their ubiquitination [55]. Syk is known to be ubiquitinated rapidly upon activation by CRP and collagen [50]. c-Cbl has no phosphatase domain but has been suggested as a necessary intermediate scaffold between Syk and its phosphatase [50]. The TULA (T-cell Ubiquitin Ligand) family of proteins are histidine tyrosine phosphatases that have been shown to be important negative regulators in immune cells [56]. TULA proteins are able to bind ubiquitinylated proteins and they play a role in regulating T-cells, decreasing Syk related protein (ZAP-70) phosphorylation [57,58]. TULA-2 exists in platelets [41,51] where it has been shown to associate with, and subsequently dephosphorylate, Syk [56]. Model B incorporates an additional Syk phosphorylation site (Y323) that is a known binding site for c-Cbl [59]. In the model, binding of c-Cbl to Y323 leads to ubiquitination of Syk, which allows TULA-2 to associate with Syk and dephosphorylate its activatory phosphorylation site (Y525), returning the receptor complex to an inactive state. All reactions are assumed to be reversible. A schematic of these newly introduced reactions is given in Fig 2, a network diagram in Fig 3 and the corresponding equations and parameters obtained from the parameter fitting process are given in S1 Text.

**Fig 5:**
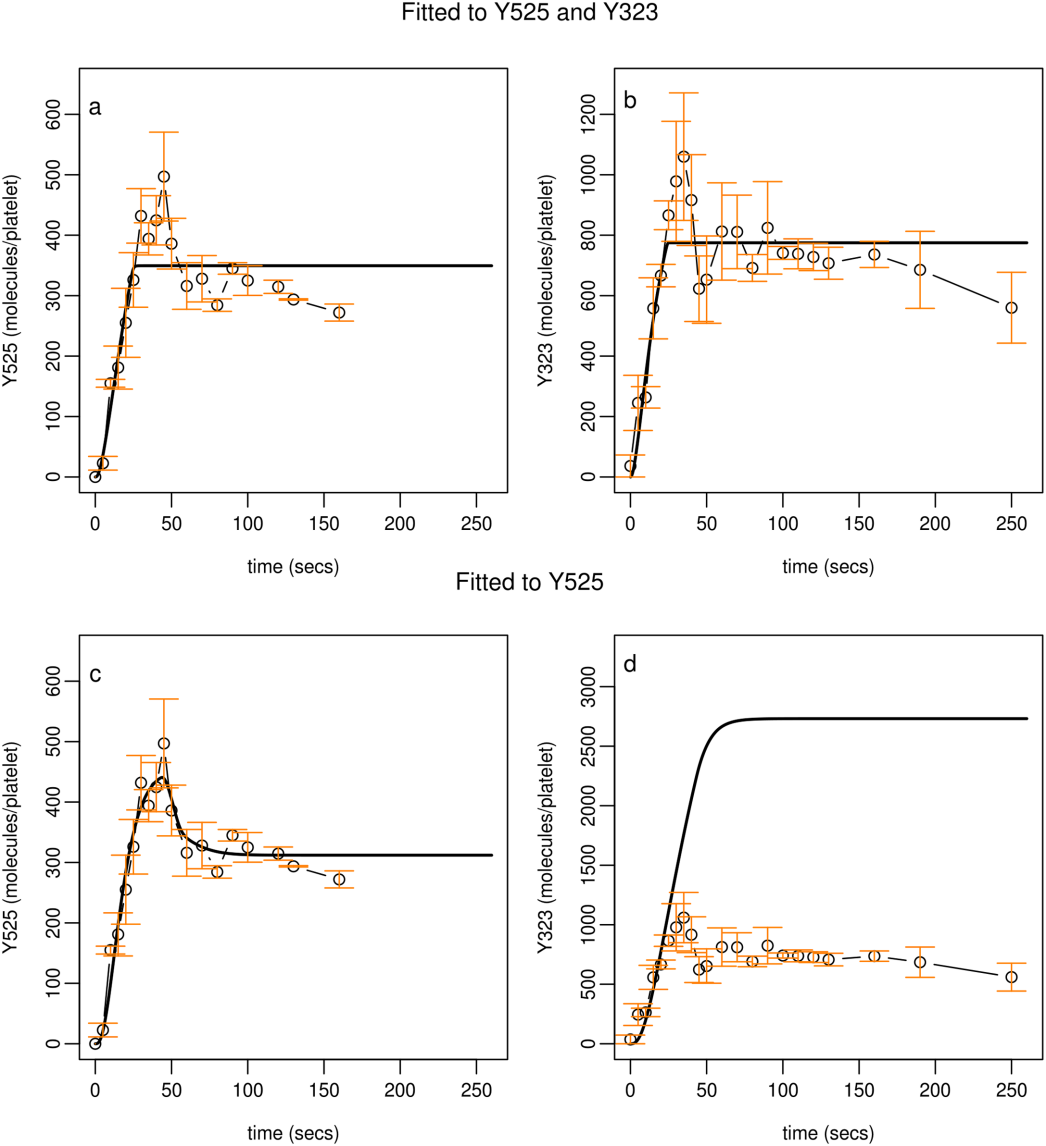
Profiles obtained from fitting Model B to experimental observations. Model profiles obtained from fitting Model B to experimental observation describing Syk phosphorylation on Y525 and Y323 (top row) faithfully represent the steady state seen in experimental data but fail to capture early transient peaks in both sets of data. Model B fitted to a restricted set of experimental observation (bottom row), describing Syk phosphorylation on Y525, closely replicates observations describing Y525 but model predictions for Y323 phosphorylation are nearly three fold higher than that seen in experimental data. In all panels experimental data of Syk phosphorylation (molecules per platelet) is the mean of three samples +/- S.E.M., phosphorylated (circles). Model simulations (solid lines) shown in panels a and b utilise parameter values from Table G in S1 Text (parameter set 1) and simulations shown in panels c and d utilise values from Table F in S1 Text (parameter set 1).

Experimental data specifically describing the newly introduced phosphorylation site was collected and Model B was fitted to both sets of experimental data simultaneously (Fig 5a and 5b). Model profiles describe accurately the steady state representing Syk phosphorylation on Y525 and the newly introduced regulatory site (Y323). They failed, however, to capture the early transient peak seen in experimental observations of both phosphorylation sites. If Model B is fitted to data restricted to one phosphorylation site (Y525) then while model solutions capture the full dynamics seen in Syk Y525 phosphorylation predictions for phosphorylation on Y323 are inaccurate, settling to a level that is three fold higher than that seen experimentally (Fig 5c and 5d).

The model steady state representing Syk phosphorylation on Y525 is given by

$$\text{Active Syk }=s_{I}-\frac{\gamma_{\text{1}}}{p_{\text{1}}}G_{\text{11}}^{r},$$ (2)

where $G_{\text{11}}^{r}$ denotes the phosphatase TULA-2 bound to Syk, and hence able to dephosphorylate it. Local sensitivity analysis (see Methods) revealed that the time to reach equilibrium was still predominantly influenced by the rate that the ITAM is phosphorylated; the level of steady state (for the activatory and regulatory phosphorylation site) is most strongly influenced by the strength of the parameters that comprise the feedback loop. The level of Syk Y525 at steady state is also sensitive to the rate of Syk phosphorylation on Y525 and its reversal. This is reflected in the change in parameter values when fitted to both sets of data; the rate of all reactions within the feedback pathway and the rate that Y525 are increased to allow the model to fit the data.

Model B accurately describes the steady states seen in the experimental observations but, though showing promise, fails to describe fully their kinetics. This led us to investigate biologically plausible refinements that would enable the model to recapture the early transitory behaviour we had seen when the model was fitted to data solely describing phosphorylation in Syk Y525.

### Model C

In formulating Model C two biologically plausible modifications to our model were explored. The first modification (H1) allows the TULA-2, when bound to the receptor complex, to dephosphorylate other Syk molecules. The second modification (H2) assumes that the increase in Syk activity (phosphorylation on Y525) increases the rate at which Y323 is phosphorylated—increases in Syk activity following Y525 phosphorylation have previously been reported [47]. The simultaneous inclusion of both modifications is denoted by Model C, H3. These reactions are depicted in Figs 2 and 3 and the corresponding equations are given in S1 Text.

**Fig 6:**
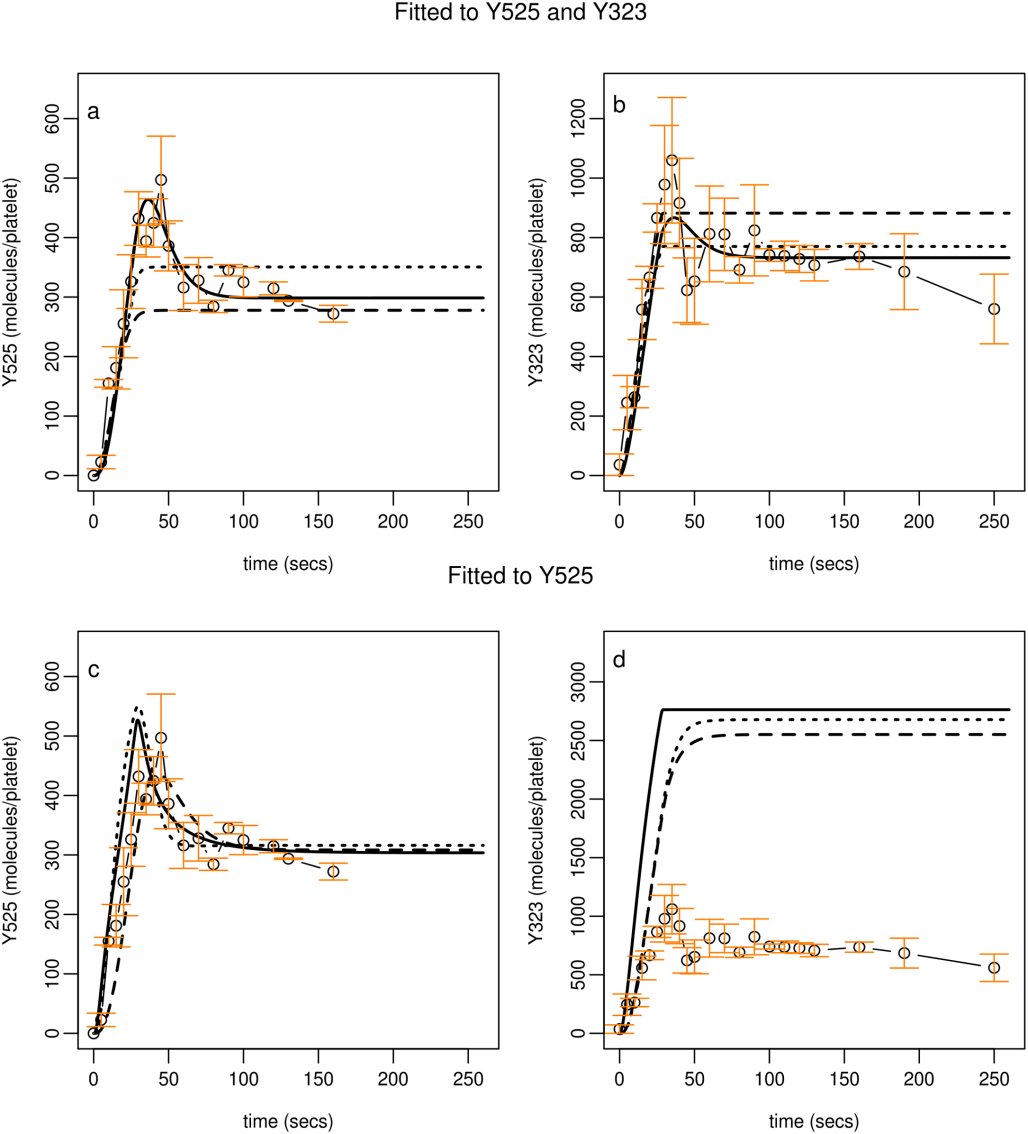
Profiles obtained from fitting Model C to experimental observations. Model profiles obtained from fitting Model C to experimental observation describing Syk phosphorylation on Y525 and Y323 are shown in panels a and b. Model C with H3 implemented (solid lines) faithfully represents the experimental data. Model C with H1 or H2 implemented (broken and dotted lines respectively) fail to describe the full dynamics displayed in the data. Model C fitted to a restricted set of experimental observation, describing Syk phosphorylation on Y525 closely replicates observations describing Y525 (panel c) but model predictions for Y323 phosphorylation (panel d) are nearly three fold higher than that seen in experimental data. In all panels experimental data of Syk phosphorylation (molecules per platelet) is the mean of three samples +/- S.E.M., phosphorylated (circles). Model simulations (H1, broken lines; H2, dotted lines; H3, solid lines) shown in panels a and b utilise parameter values from Table J in S1 Text (parameter set 1) and simulations shown in panels c and d utilise values from Table I in S1 Text (parameter set 1).

**Fig 7:**
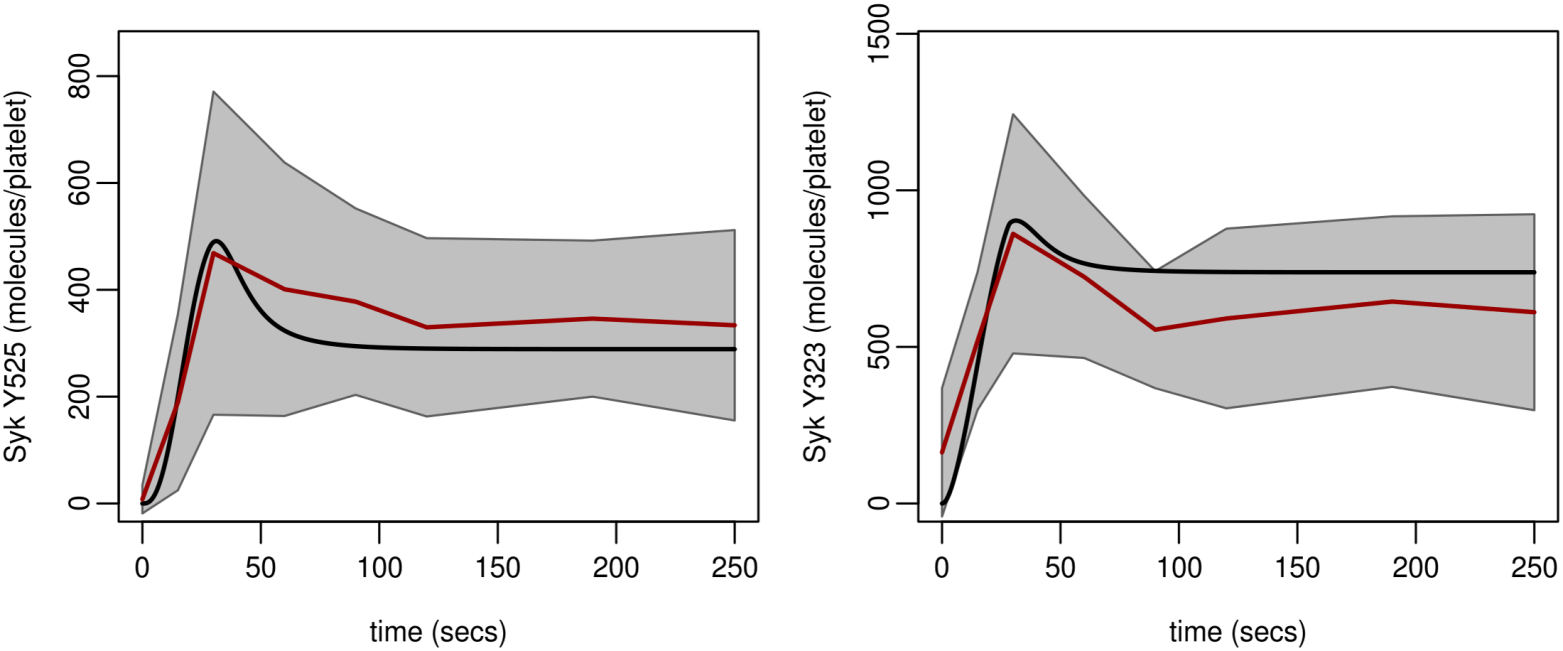
Model profiles compared to experimental data from taken from nine donors. Experimental values are mean (solid red line) +/- 95% C.I. (shaded region) and of all data. Model profile (solid black line) utilises Model C (H3). Parameter values and initial conditions are taken from Tables J (parameter set H3,1) and D in S1 Text respectively.

Model C, H1 and H2 profiles (Fig 6a and 6b broken and dotted lines respectively) fail to describe transitory behaviour in both sets of experimental data. Model C, H3 (Fig 6a and 6b, solid line) incorporates H1 and H2 simultaneously and is able to describe accurately both sets of experimental observations. These optimal fits were dependent on the models being simultaneously fitted to both sets of experimental data. Like Model B, if fitted to one set of observations (i.e. Y525), model predictions for Y323 were inaccurate (Fig 7c and 7d).

The steady state that model profiles representing Syk phosphorylation on Y525 is determined by

$$\text{Active Syk }=s_{I}-\frac{\gamma_{\text{1}}}{p_{\text{1}}}G_{\text{11}}^{r}-\left(\text{TULA-2 bound}\right)\left(G_{Y525}-G_{\text{11}}^{r}\right),$$ (3)

where *G*_*Y525*_ denotes the portion of Syk molecules that are bound to the receptor complex and phosphorylated on Y525. Local sensitivity analysis (see Methods) revealed (see Fig G in S1 Text) that the steady state is influenced by parameters that comprise the whole regulatory pathway reflected in the above by the proportion of TULA-2 that is bound to the receptor complex. In agreement with Thomas et al [51] the above expression (3) and sensitivity analysis (see Fig J in S1 Text) confirm that a decrease in the levels of TULA-2 lead to hyperphosphorylation of Syk. The time to reach the above steady state rate is shown to be predominantly influenced by the rate of ITAM phosphorylation while the time to reach the steady state of the regulatory phosphorylation site (Y323) is influenced by the rate of ITAM phosphorylation and by protein copy numbers of GPVI and Syk. Of the parameters that were held fixed during the parameter fitting process (platelet volume, extracellular volume and rates of ligand binding and dissociation) only the platelet volume influences Syk activity.

In summary, the ability of the model to describe the full range of dynamics seen in the experimental observations requires that Syk activity increases the rate of phosphorylation on Y323 and that TULA-2 is able to dephosphorylate not only the receptor to which it is bound but also any nearby receptor complex.

### Model selection, validation and predictions

To understand if the extension of each model is justified through a better fit to data we use the Akaike information criterion (AIC) and a modified form that takes into account small experimental sample sizes (AICc)—a description of the metric and how we apply it is given in Methods and the results are shown in Table 3. The model with the lowest AICc provides the best balance between its replication of data and the added complexity introduced to achieve it.

**Table 3:** Model comparisons based on the Akaike’s Information Criterion (AIC) and its corrected form (AICc).

Model	*K*	*n*	SSE	AIC	AICc
A	4	22	6.65e4	184.22	**186.57**
B	12	22	**2.61e4**	**179.72**	214.38
C, H1	11	22	3.08e4	181.37	205.37
C, H2	11	22	2.79e4	179.19	203.19
C, H3	12	22	2.85e4	181.66	216.32
B	12	44	3.56e5	419.93	429.99
C, H1	11	44	3.78e5	420.57	428.82
C, H2	11	44	3.84e5	421.26	429.51
C, H3	12	44	**2.63e5**	**406.61**	**416.67**

K = number of model parameters fitted to data, n = number of experimental observations (22 observations of Syk Y525 and 44 of Syk Y525 and Y323), SSE = distance from experimental observations. Models with the lowest AICc are the most likely, but only when compared to models fitted to the same value of n. Metrics in bold denote the lowest SSE, AIC or AICc for a given set of experimental observations.

Model C (H3) has the lowest AICc; it provides the most faithful replication of both sets of data (a total of forty-four experimental observations) and is not overly compromised by its additional complexity when compared to Model B. If data are restricted to that describing one phosphorylation site (Y525, twenty-two time-points) then Model A has the lowest AICc; Model B and C are too complicated (too many parameters) to be inferred from such a set of observations. Overall, these results confirm Model C, H3 as our preferred model and this was used for all further simulations.

#### Model simulations compared to data from a wider healthy population of donors

While the model was calibrated to data based on platelets taken from a single donor, within a wider healthy population a platelet response to agonist stimulation can show substantial inter-donor variation. This variation may be attributed to environmental influences, age, sex, diet, medication, stochastic variation in protein expression levels and/or genetic factors [60–64]. With a model established, simulations were compared to data from nine additional healthy donors. Following stimulation with CRP, levels of Syk phosphorylation (Y525, Y323) were measured over a period of two hundred and fifty seconds and quantified as described above. Fig 7 demonstrates that model outputs compare well to the mean of experimental observations observed within this population. The model profiles are close to the mean and within the 95% confidence interval of the mean indicating that the calibration of the model to the initial blood donor was representative of the wider healthy population.

#### Participation of regulatory proteins

**Fig 8:**
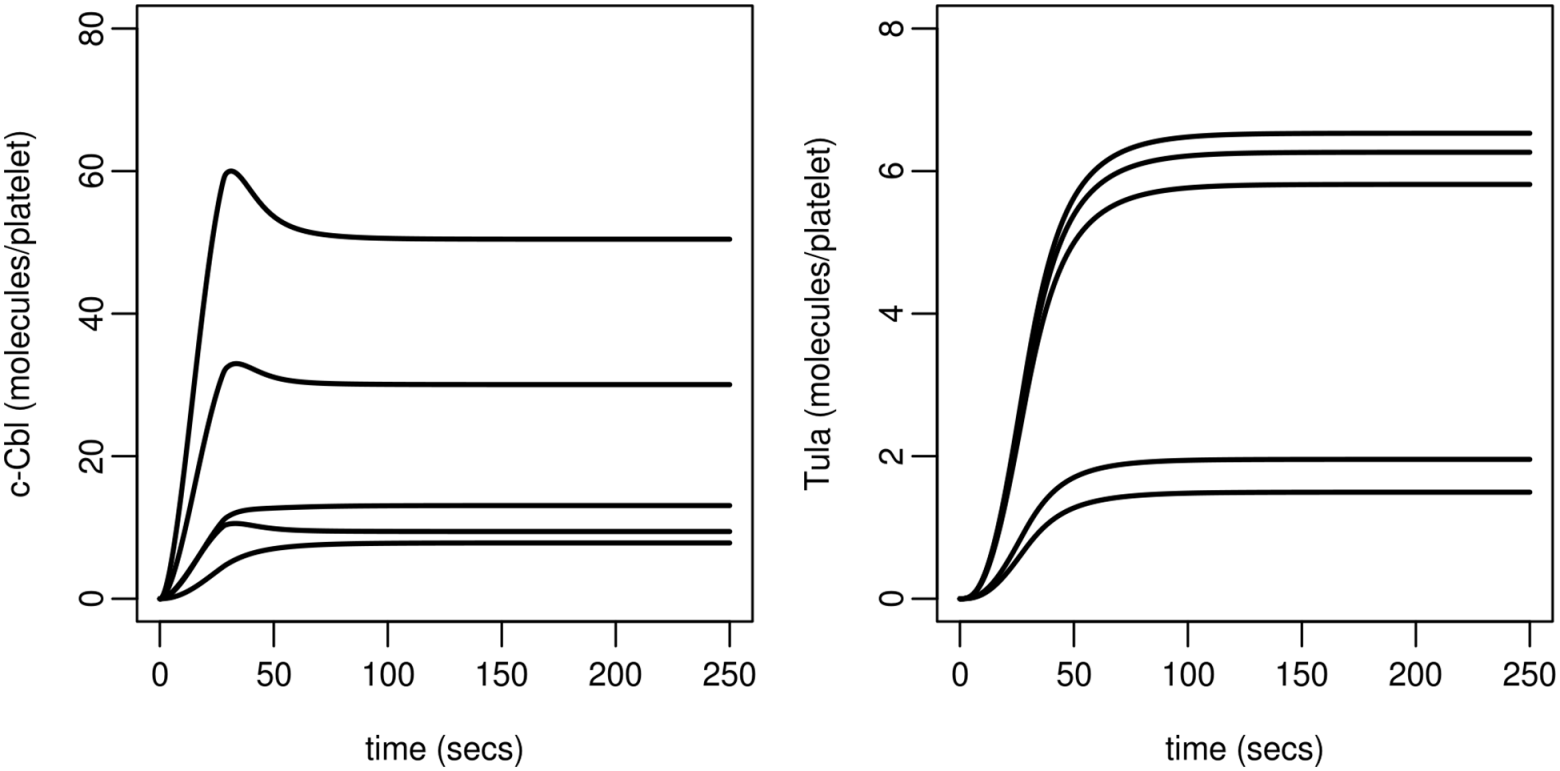
Model predictions for regulatory protein participation. Simulations utilise the five best parameter sets (Table J in S1 Text) obtained from fitting the final model (i.e. Model C, H3) to experimental data: the variation in model predictions is low and all simulations predict that very few of the regulatory proteins, c-Cbl and TULA-2, are required to control Syk activity.

The development of the model required us to incorporate recruitment of TULA-2 to be able to influence the dephosphorylation of additional Syk molecules within the environment into which TULA-2 is recruited (Model C, H2). GPVI receptors are thought to be localised in lipid rafts (glyco-phospholipid micro-domains within the plasma membrane) [65]. The clustering of receptors would result in the ability of termination of the signal to be propagated to surrounding activated receptors. We therefore hypothesised that the levels of c-Cbl and TULA-2 required for regulation of the trajectory of the GPVI pathway may be lower than that originally anticipated based upon the levels of phosphorylated Syk. Simulations of the model (Fig 8) demonstrate that on average the levels of c-Cbl and TULA-2 required to modulate Syk activity are in fact very low (c-Cbl: between 10 and 60 on average within a population, per platelet; TULA-2: approximately one order of magnitude lower).

#### Model simulations compared to data describing the effects of ligand depletion

**Fig 9:**
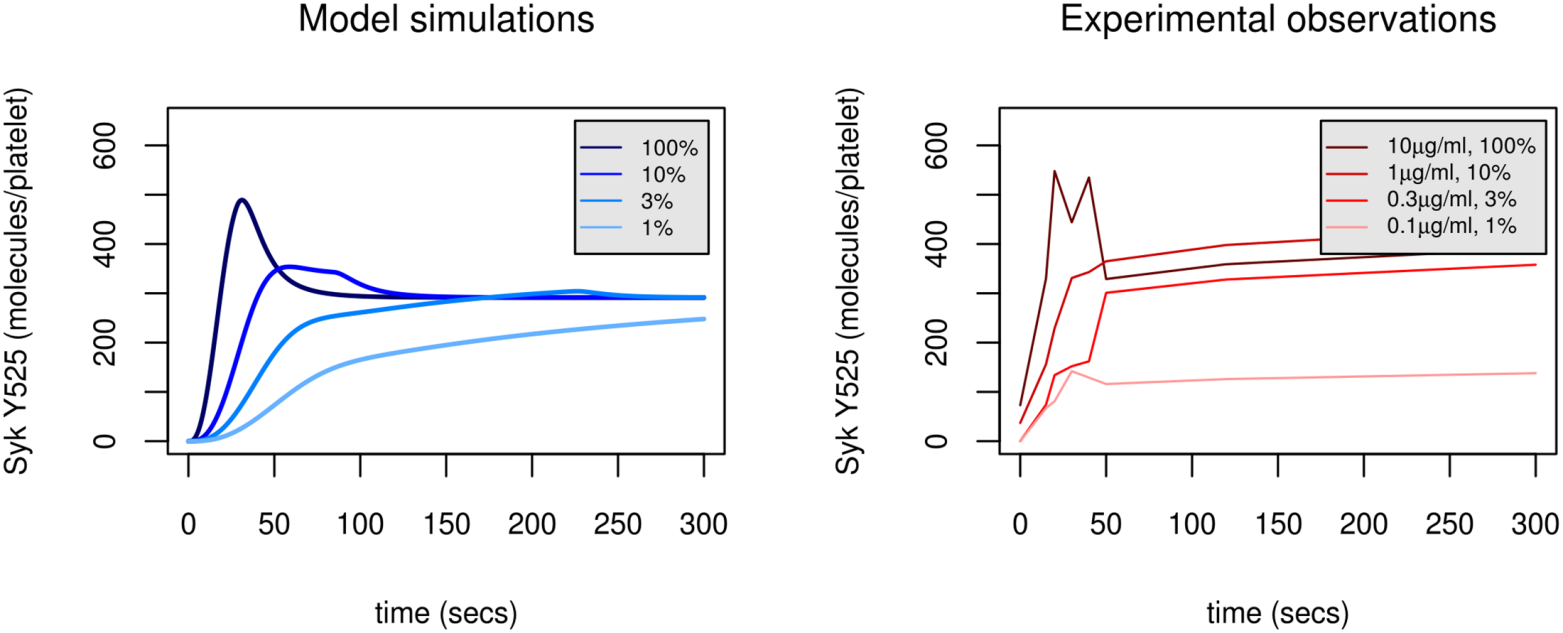
Model predictions of the effect of ligand depletion (left) show close agreement to experimental data (right). Model profiles of Syk activity are the result of a series of simulations where the ligand is decreased from 100% through to 1% of its original value. Experimental observations (right) are the result of a series of experiments where the agonist (CRP) is decreased from 100% through to 1% of its original value. Model profile (solid black line) utilises Model C (H3). Parameter values and initial conditions are taken from Tables J (parameter set H3,1) and D in S1 Text respectively.

Collagen or collagen related peptides are unusual ligands, in that they are polymeric in nature and able to bind multiple collagen receptors, resulting in receptor clustering and the initiation of cell signalling [66]. Since the precise concentration of physiological ligand is hard to assess as platelets would be expected to encounter a surface of collagen potentially resulting in exposure to saturating levels of the ligand, the model incorporated the assumption that ligand was unlimited. Since this assumption may not be correct, it was important to determine the ability of the model to predict the effect of ligand depletion on the modulation of Syk activity. The effects of reducing CRP concentration by two orders of magnitude was therefore measured over eight times point within a period of three hundred seconds and compared with model simulations. The model was able to provide a close prediction of experimental levels of activated Syk (Y525 phosphorylation) in response to stimulation by a decreasing series of concentrations of the ligand (from 10μg/ml (100%) to 0.1μg/ml (1%), Fig 9). Both model profiles and experimental data showed an initial loss of the early transient peak in phosphorylation and a delay to reach the steady state as the ligand concentration was reduced. Larger decreases (99%) in concentration of the ligand resulted in a significant delay in Syk phosphorylation such that (over the timespan of interest) Syk activity was reduced.

#### Impact of population variability

**Fig 10:**
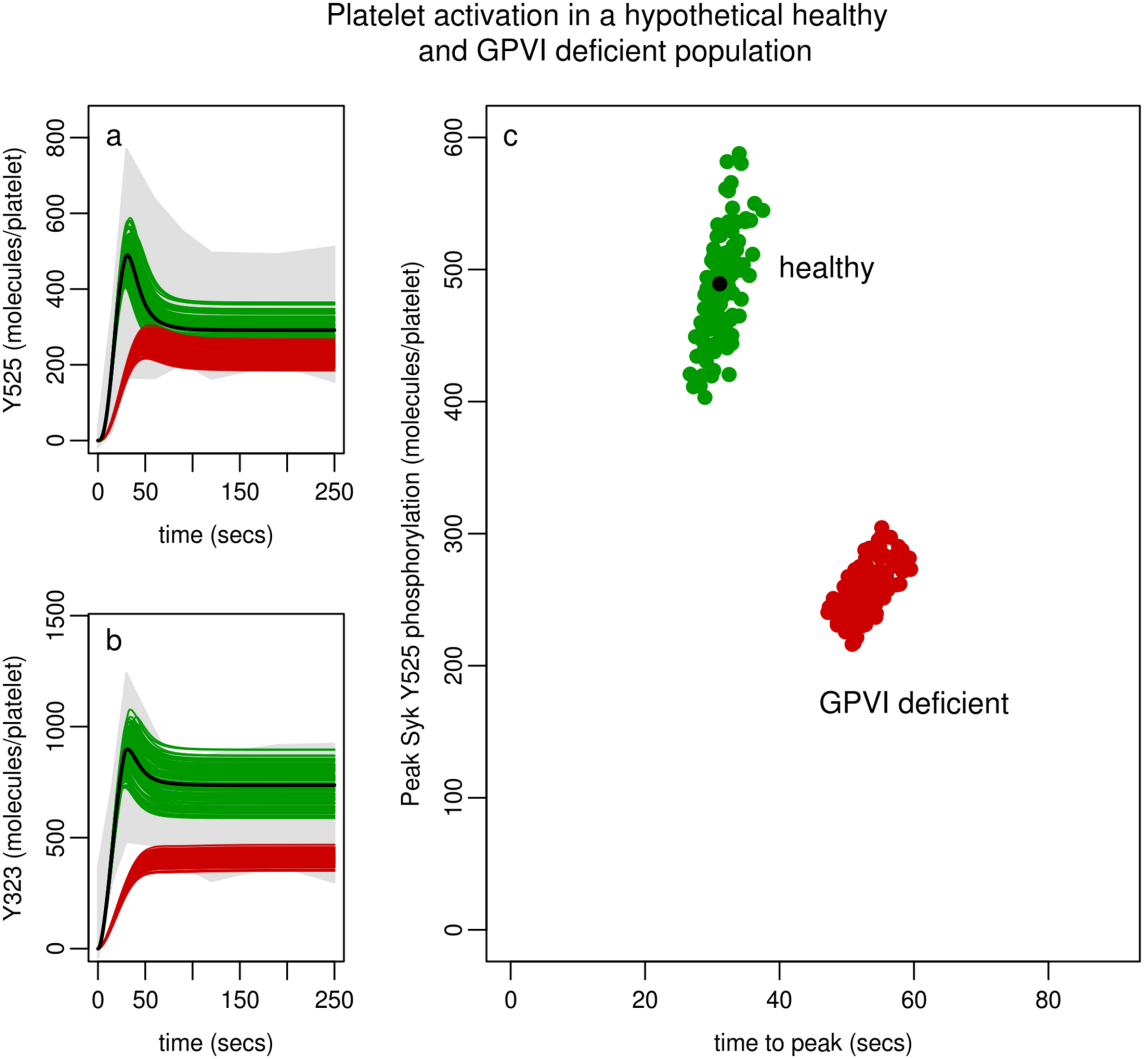
Syk activity in a hypothetical population. The results of 200 simulations are shown (each simulation representing a hypothetical donor). Two markers of each simulation (peak Syk Y525 phosphorylation and time-to-peak) are denoted graphically by a circle. Two distinct populations are shown: a healthy population (green) where protein composition were varied across their normal range and a population with 75% deficiency in levels of GPVI. In all simulations parameter values are held fixed to those shown in Table J in S1 Text, set H3,1. The initial conditions of GPVI, Syk and c-Cbl are randomly selected from a range (healthy population: GPVI, +/-12%; Syk +/-22%, c-Cbl +/-25%; GPVI deficient population: GPVI, -25%+/-12%; Syk +/-22%, c-Cbl +/-25%) from the values given in Table D in S1 Text while TULA-2 is held steady to the value shown as there is no variation data for this protein. The large black circle indicates an individual with the mean protein levels that were obtained experimentally (GPVI, 5000; Syk, 2763; c-Cbl, 2581).

Whilst variability of platelet responses to collagen within the population are not fully understood, common polymorphisms in GPVI genotype are known to impact on GPVI expression levels. We therefore sought to explore the effects of variations in copy numbers of key proteins within the model in the context of varying levels of GPVI. This included analysis across the normal range of GPVI expression within the population, and also in the context of the levels of deficiency (25% (and the normal variability around this starting point)) that have been shown to be associated with specific gene mutations in GPVI [67,68]. Simulations show two populations: (I) a healthy population in which levels of GPVI, Syk and c-Cbl are known to vary (by +/-12%, +/-22% and +/-25% respectively) and (ii) a population where GPVI is deficient (by 25%). In simulations representing 100 hypothetical healthy donors the three protein copy numbers were allowed to vary from the optimum obtained from the fitting process, taking random values from a range seen in our experimental data, or in the case of GPVI that are reported in the literature [41], to occur in healthy individuals (GPVI, +/-12%; Syk +/-22%, c-Cbl +/-25%). In simulations representing 100 hypothetical GPVI deficient donors Syk and c-Cbl protein copy numbers were allowed to vary as they were in the healthy population but in the case of GPVI variation (+/-12%) was based on a 25% deficiency from the base value used for the healthy population. Fig 10c shows the range in two markers of Syk activity (peak Y525 phosphorylation and time to reach peak) for each hypothetical donor while Fig 10a and 10b shows the time-dependent predictions of Syk phosphorylation, for each hypothetical donor, compared to Syk activity, and regulation, as seen in a healthy population of donors (as per Fig 7). Model simulations demonstrate that while intra donor variation in expression levels can contribute to much of the variability in peak Syk activity, the time to reach this peak is tightly controlled. The deficiency in GPVI expression levels that occur in 15% of the population result in a 50% decrease in peak Syk phosphorylation, and the ability of the receptor to signal downstream, and a delay in the time to reach this peak.

## Discussion

A major success over the last 20 years has been the identification of the key components of the major signal transduction pathways in cells [2–7]. What is less well understood is how these components come together in space and time in a regulated manor to bring about appropriate cellular responses. Whilst the activation of protein kinases has been studied in some detail, much less work has been done on the regulation of phosphatases in part due to the lack of specific inhibitors. A search of the literature shows that there are 20 times the number of papers on protein kinases in platelets as there are on protein phosphatases. However as noted earlier most cells, including platelets, contain many different types of protein-tyrosine phosphatases (there are roughly the same number of different protein-tyrosine phosphatases as there are protein tyrosine kinases in platelets [40]), that show distinct subcellular compartmentalisation and regulation. Some appear to be simple abundant cytosolic phosphatases without obvious regulatory domains which may be constitutively active, some are transmembrane receptor type phosphatases such as CD148, whilst others are highly regulated containing domains that allow them to be temporally recruited to specific signalling complexes either via SH2 domain phosphotyrosine interactions (eg., SHP1 and SHP2) or through ubiquitin-dependent binding (TULA-1 and TULA-2) [40–45]. Thus the challenge now is to determine which of these phosphatases are required and sufficient to explain the regulation of the key determining steps in individual signalling cascades. The objective was to develop a mathematical model of GPVI proximal signalling that allows the comparison of current biological knowledge and hypotheses to high-density temporal experimental data explicitly collected to inform the model. The experimental observations describe in particular phosphorylation of Syk, a key protein that is recruited to the GPVI receptor and which initiates downstream signalling. It was anticipated that this approach would lead to increased knowledge surrounding the interaction of the components of this key step in platelet activation and, importantly, how this is controlled.

The model was developed incrementally. Starting with a simple model (A) that captured current biological knowledge of the components and interactions that occur just downstream of the GPVI receptor more complicated interactions were incorporated until a good description of experimental data was achieved. While the components that comprise the forward steps of platelet signalling have been well studied, regulatory processes have not. The experimental data pointed to the pathway being regulated and, in consequence, a simple housekeeping phosphatase that limits the ability of Syk (and therefore the receptor) to signal downstream was incorporated into Model A. The model was unable to explain the full dynamics seen in experimental observations and led us to conclude that the inclusion of a simple phosphatase, despite their abundance in platelets [40], is insufficient to explain data. A potential regulatory pathway was selected, supported by current literature, which focuses on the Syk protein and its ability to participate in its own regulation through proteins c-Cbl and TULA-2 [49–55]. Our initial attempts to explain the experimental data while promising did not capture the full range of dynamics displayed in observations of the central protein Syk on a phosphorylation site that we equate to activity and one that initiates the ability of Syk to regulate itself [46–47]. With further model refinements (Model C) we found that a model with c-Cbl/TULA-2 incorporated could provide not only a good quantitative fit to experimental data but could capture its full dynamics. As demonstrated in sensitivity analysis the inclusion of the negative feedback loop protects Syk activity from variation in the number of Syk molecules.

Interestingly, if the experimental data is limited to that describing a single Syk phosphorylation site (Y525) then the inclusion of the more complex regulatory mechanism (centring on c-Cbl and TULA-2) cannot be justified. It is only when data describing a second phosphorylation site was collected that Model B and Model C could be successfully differentiated. This emphasises the need for high density data to allow the development of such complex models that incorporate more subtle processes (and many parameters).

The model predicts that, on average, very few molecules of c-Cbl and TULA-2 need to be bound to the receptor complex at any point in time. This in turn suggests a significant stochastic component to the behaviour. This, and the prediction that regulation of activated Syk molecules relies on the ability of the bound phosphatase, TULA-2, being able to dephosphorylate many activated Syk molecules, point to receptors being clustered at the plasma membrane. This fits well with the localisation of GPVI to specific membrane domains and the known ability of the multivalent ligand, collagen, to drive localised clustering of GPVI at the platelet surface [65] but also highlights the importance of further spatial mathematical investigations.

To be of wider use it is essential that the model can accurately predict experimental signalling outputs under varying conditions. The model was tailored to an environment where the ligand is abundant but is able to predict Syk activity in response to decreasing levels of ligand availability. A decrease in the availability of the ligand in the model results in a reduction of the early transient peak and a delay to reach the steady state which shows good agreement with independently generated experimental data.

Platelet signalling is likely to demonstrate a wide degree of variation across a healthy population but little is known about the extent and at what point this leads to potential pathologies. This normal variation is seen in the levels of the individual proteins involved in our model, both in our data and the recent quantitative human platelet proteome [40] and also in the signalling responses of individual donors studied experimentally (Fig 7). In the first instance the model was fitted to data based on triplicate samples from one healthy donor. By comparison to data describing Syk phosphorylation, on both the activatory (Y525) and regulatory (Y323) sites, in nine additional donors we found the model was able to replicate phosphorylation profiles observed in a wider healthy population. The model was also utilised to investigate signalling responses in a population that has reduced levels of GPVI receptors [1,2] with normal population variability of expression levels of GPVI and other signalling components involved (Fig 10). This suggested that the time to peak of Syk activation in normal individuals seems to be tightly controlled whilst the actual peak maxima of Syk Y525 phosphorylation is more variable, as was also seen experimentally in our normal panel of nine healthy donors who all had very similar times to peak but quite variable absolute levels of Syk Y525 phosphorylation (Fig 7). The simulated population with reduced GPVI levels however had a longer time to reach this peak and the timing seems to be less tightly controlled within the simulated population. This may suggest that temporal regulation of this signalling pathway may be more important to a functional response than the absolute magnitude of the response.

In summary, this study aims to bridge the gap between biological knowledge and hypotheses and temporal experimental data in aiding understanding of how the components of a key initiating step in platelet activation interact to control precisely a platelet's response to its environment. Platelet activation has traditionally been thought of as a series of forward steps resulting in the platelet switching on. While the fine details of even this initiating step will continue to be elucidated, the principal of negative pathways playing a central role is clear and highlights the complexity of these signalling pathways. Indeed there is evidence of a number of inhibitory receptors and signalling mechnisms including those stimulated by nitric oxide and prostacyclin that serve to prevent un-required activation [69]. Some mechanisms such as those stimulated by the adhesion receptor PECAM-1 are known to modulate signalling downstream of the processes explored in this study, although we cannot rule the potential impact of these mechanisms on the initiation of GPVI signalling. Given the outcomes of this study, this will be the focus for future work. While our approach here has focused on elucidating a key regulatory step in a platelet signalling pathway we believe that this approach of integrating biological knowledge with time-rich kinetic data sets and mathematical models is well suited to the analysis of the regulation of any signalling cascade for which suitable reagents are available.

## Methods

### Experimental techniques

Here we briefly describe recently established methods utilised for the measurement and quantification of protein copy numbers and their post translational modifications. Please see [70] for further details.

The quantification method is based on the relationship between a known amount of a specific protein to a quantitative signal emitted by fluorescently conjugated antibodies. Individual phosphorylated proteins were first isolated by immunopreciptation (IP) using suitable site-specific antibodies. Identical serial dilutions of the IP were loaded onto two SDS-PAGE gels. One gel was also loaded with a serial dilution of a known concentration of a corresponding non-phosphorylated recombinant protein. The resulting immunoblot was probed with an antibody recognising the total protein (i.e. non phosphorylated). The second gel loaded with the IP serial dilutions was blotted then probed with the same phospho-specific antibody used for the IP. Both gels also contained serial dilutions of known amount of IgG necessary to calibrate with subsequent experiments. The different immunoblots were then treated with appropriate fluorescent conjugated secondary antibodies and scanned with a fluorescence imaging system. The resulting values for the recombinant protein dilution were then used to construct a standard curve and determine the concentration of molecules immunoprecipitated. Combined with the results from the second immunoblot allows the direct relationship between the amount of protein actually present on the immunoblot and fluorescent levels obtained when probing with a phosphospecific antibody to be determined. Further experimental samples were loaded on SDS-PAGE gels together with identical IgG serial dilutions for normalisation with the reference datasets and quantification of the amount of phosphorylated proteins. The amount of total protein was established by comparing the normalised fluorescence intensity of the samples and the recombinant protein standard curve when probed with the antibody recognising the total protein.

Blood samples were obtained from healthy volunteers that had given consent, using procedures approved by University of Reading Research Ethics Committee. Washed platelets were prepared by differential centrifugation, as described previously [70], and resuspended in Tyrodes buffer containing 0.4U/ml Apyrase,1mM EGTA and 100 μM Indomethacin; to suppress secondary signalling and secretion. Samples were stimulated with CRP-XL (provided by Prof Richard Farndale, University of Cambridge, UK) at a final concentration of 10 μg/ml, then lysed, denatured and loaded onto 10% SDS-PAGE gels. CRP-XL, unlike collagen, is a GPVI selective agonist. Reference datasets were constructed using platelets treated with pervanadate [71] (10 μg/ml) CRP for 1 min. Immunoprecipitation was carried out using PureProteome Protein A magnetic beads (Millipore). Immunoprecipitates and experimental samples were loaded alongside the corresponding recombinant protein (Syk, GST-tagged, Abnova; c-CBL proprietary tag, Abcam) and a series of dilutions of IgG (Rabbit and Murine Isotype controls, US Biological). Westernblot transfer to Immobilon-FL membrane (Millipore) was performed using a semi-dry blotter (Bio-Rad). The membranes were blocked with 5% (w/v) BSA and probed as instructed by the manufacturer using the Millipore SNAP i.d. protein detection system and the appropriate antibodies (Anti-Syk N-19, Santa-Cruz Biotechnology Inc; anti-c-Cbl, BD Biosciences; anti-Syk phospho Y525+Y526, anti-c-Syk Y323). Immunoblots were treated with either a fluorescent Cy5 dye-labeled goat anti-Rabbit or a Alexa-Fluor 647 dye-labeled donkey anti-mouse (Life Technologies). Digital scan values of the fluorescence emission were obtained using a Typhoon Trio scanner (GE Healthcare). Quantification was performed using the ImageQuant TL software and data analysed using R [72].

### Parameter estimation

We estimated all unknown parameter values for our models by utilising a constrained local optimization routine (MATLAB's fmincon) that varies all unknown parameters to minimize the differences between the model and experimental data via a cost function

$$\text{Sum Squared due to Error }(\text{SSE})={\left(y_{i}\left(\theta \right)-Data_{i}\right)}^{\text{2}}$$ (4)

where *y*_*i*_ *(θ)* is the model’s prediction for the relevant model variable (which depends on the parameters *θ*) and Data_i_ represents the experimental observations at time points *i*. Given two sets of parameters, the one with the smaller cost function is the one that provides the better description of experimental observables. The values that parameters can take are restricted within limits that are guided by biological knowledge and literature (S1 Text includes a summary of the limits used and how they were set).

The optimization algorithm takes steps that successively decrease the value of the cost function, beginning from an initial guess of the parameter values. To avoid parameter sets being selected that fit local, rather than global, minima we utilize a multi-start approach where repeated optimization runs are carried out with multiple initial guesses. We sampled initial guesses from the complete allowable parameter range utilising Latin Hypercube Sampling (LHS). The range that parameter values can take is discretised into N bins. Where parameter limits extend several orders of magnitude, a logscale is used, which leads to a better representation of parameter space. Each interval in the parameter range is sampled once (without replacement), so that the entire range for each parameter is explored. The resulting N samples are used as initial guesses. Parameter estimation is performed utilising samples N = 1000. The five parameter sets with the lowest SSE N = 1000 are reported in each case to provide confidence that the best fit is converging, for the final model (C, H3) samples of N = 10000 are reported, and needed to converge.

### Parameter sensitivity analysis

We performed a local sensitivity analysis, where each parameter was varied by fifty and ninety percent above and below their initially estimated value, and computed the normalised local sensitivity of either the final steady state or the time to reach this steady state according to

$$\text{Sensitivity Score }=\frac{\left(O_{a}-O_{i}\right)}{O_{a}}$$ (5)

where *O*_*i*_ and *O*_*a*_ represent the model output (e.g. the steady state and time to reach the peak in Syk activity) in respect of the initial parameter set (obtained from parameter fitting) and the adapted parameter respectively.

### Model comparison

To aid model comparisons we used Akaike’s Information Criterion (*AIC*) [73]. This is an information-theoretic criterion for model comparison, which incorporates not only the cost function value from the parameter fitting process (*SSE*) but also a penalty based on the number of parameters (*K*) in the model. We used a modified criterion [68] that takes into account the experimental sample size (*n*) by increasing the relative penalty for model complexity with small datasets defined by

$$AIC_{c}=AIC+\frac{\text{2K}\left(K+\text{1}\right)}{\left(n-K-\text{1}\right)}$$ (6)

where

AIC=n(ln(SSE/n))+2K. (7)

*AICc* converges to *AIC* as n becomes large with respect to *K*. The value of *AICc* has no meaning in isolation, its relevance only becoming apparent when it is used to compare (and rank) models fitted to the same experimental data. In general a better fit to experimental data is achieved when the complexity of a model is increased, simply because the number of free parameters increases. The *AICc* (and indeed the *AIC*) aims to protect against this by a penalty based on the number of parameters in the model with the lowest *AICc* being the better model.

## Supporting Information

S1 Text Details of the mathematical models, their parameterisation and sensitivity analysis. (PDF)
