# Supplementary material for: Regulation of Early Steps of GPVI Signal Transduction by Phosphatases: A Systems Biology Approach
Source: PLoS Comput Biol. 2015 Nov 19;11(11):e1004589. doi: 10.1371/journal.pcbi.1004589 (PMC4652868; doi:10.1371/journal.pcbi.1004589)
Supplement: S1 Text — (PDF) [file pcbi.1004589.s001.pdf]

SUPPORTING INFORMATION TEXT S1 FOR **Regulation of early steps of GPVI signal transduction by phosphatases: a systems biology approach.**

J.L. Dunster, F. Mazet, M.J. Fry, J.M. Gibbins and M.J. Tindall

We present details on all three mathematical models. Each model describes the same subcellular events that follow a ligand binding to a platelet's GPVI receptor, but vary in their method of regulation.

S.1 Model A

In each of our models a ligand, CRP, (denoted by l) binds to a platelet's GPVI receptor (g) to form a receptor complex (G). Binding of the ligand directly leads to phosphorylation of the receptor complex (G^p) allowing the cytosolic protein tyrosine kinase Syk (s) to dock (G_0^b) and subsequently phosphorylate (G_1^b). We equate Syk phosphorylation to its activity state and the subsequent ability of the receptor to signal downstream. In Model A regulation of Syk activity is incorporated in the form of a simple, constituitevely active, abundant phosphatase that can dephosphorylate Syk. A network diagram representing the events incorporated in Model A is shown in Fig. 3 (upper panel) while a summary of the variables and dimensional parameters is presented in Tables A and B, respectively. Utilising mass action kinetics these reactions transform to give the following system of nonlinear ordinary differential equations (ODEs)

$$\frac{dg}{dt} = -k_1\, g\, l + k_{-1}\, G, \tag{S1a}$$

$$\frac{dG}{dt} = k_1\, g\, l - k_{-1}\, G - k_2\, G, \tag{S1b}$$

$$\frac{dG^p}{dt} = k_2\, G - \frac{k_3}{V_p A_v}\, G^p\, s, \tag{S1c}$$

$$\frac{dG_0^b}{dt} = \frac{k_3}{V_p A_v}\, G^p\, s - p_1\, G_0^b + \gamma_1\, G_1^b, \tag{S1d}$$

$$\frac{dG_1^b}{dt} = p_1\, G_0^b - \gamma_1\, G_1^b, \tag{S1e}$$

$$\frac{dl}{dt} = -\frac{k_1}{V_e A_v}\, g\, l + \frac{k_{-1}}{V_e A_v}\, G, \tag{S1f}$$

$$\frac{ds}{dt} = -\frac{k_3}{V_p A_v}\, s\, G^p, \tag{S1g}$$

where A_v represents Avogadro's number, V_e the extracellular medium per cell available under experimental conditions and V_p the cytosolic volume per platelet. These parameters ease conversion of reactions that occur between proteins that are embedded in the cell surface (receptors) and those that are in the extracellular space or the cell's cytosol. To facilitate comparison to experimental data the variables representing the receptor, in all its forms, and cytosolic proteins are expressed in molecules while the ligand is expressed as a concentration.

The initial conditions prior to ligand addition are set so that

$$g = g_I, \qquad G = 0, \qquad G^p = 0, \qquad G_0^b = 0, \qquad G_1^b = 0, \qquad l = l_I, \qquad s = s_I. \tag{S1h}$$

i.e. the signalling cascade is inactive, all receptors are unbound and all ligands free.

Conservation of the ligand, receptor and Syk is given by

$$l_I - l = \frac{1}{V_e\, A_v}(G + G^p + G_0^b + G_1^b), \qquad g_I - g = G + G^p + G_0^b + G_1^b, \qquad s_I - s = G_0^b + G_1^b, \tag{S2}$$

respectively.

S.1.1 Parametrisation

Here we describe the model's parameters and initial conditions that are either well defined in the literature or we have been able to determine experimentally (initial conditions and parameter values are summarised in Tables A and B).

The rates that the ligand, CRP, binds to and dissociates from, a GPVI receptor are given in [10], allowing us to set $k_1 = 8$ m^3 moles^{-1} s^{-1} and $k_{-1} = 3.02 \times 10^{-2}$ s^{-1}. We obtained the initial conditions for the number of GPVI receptors and Syk molecules per platelet experimentally so that $g_I = 5000$ molecules and $s_I = 2763$ molecules. Determining the initial conditions for the ligand was complicated by CRP being of an indeterminate length, weight and number of binding sites for a GPVI receptor. This made it difficult to convert from the experimental units of mass concentration (measured in units of μg/mL) to the model's molar concentration (measured in units of moles/m^3). In all of our experiments we utilised a high saturating level of the ligand (10μg/mL) and therefore for our model estimated $l_I = 3 \times 10^{-2}$ moles m^{-3}, a level that is non-rate limiting. For every blood sample taken we measured the mean platelet volume allowing us to set $Vp = 7.4 \times 10^{-18}$ m^3 and in all experiments platelets were suspended in media at a concentration of 3×10^8 cells/mL [2] giving us an extracellular volume per platelet (Ve) of 3.33×10^{-9}m^3.

S.1.2 Parameter fitting

We obtained values for the remaining parameters (k_2, k_3, p_1, γ_1) via a process of parameter fitting that is described in the main paper (see Methods). The values that these parameters could take are constrained to biologically realistic limits. These are based on estimates in the literature, for similar processes either involving different proteins and/or in different cell lines.

Estimates describing phosphorylation are reported in the literature to be in the range $0.1-100$ s^{-1} [3,5,11]. Relaxing this range by one order of magnitude we allowed parameter values for phosphorylation to fall between a range of $10^{-2}-10^{3}$ s^{-1}. Binding rates for proteins are reported to be in the range of $1\times10^{2}-1\times10^{5}$ m^3 moles^{-1} s^{-1} [5–8,11,12] allowing us to set a range of $10^1 - 10^6$ m^3 moles^{-1} s^{-1} for all protein binding. The reverse processes of dissociation and dephosphorylation are reported to be in the range $0.03 - 20$ s^{-1} [5,7,8,11,12] allowing us to set a range of $1 \times 10^{-3} - 10^{3}$ s^{-1}.

S.1.3 Model A solutions

Table C provides the five 'best' (lowest SSE) results of fitting Model A to experimental observations of Syk phosphorylation on Y525 (utilising a sample of $N = 1000$ different initial guesses). The parameter sets that are described only represent a sample of those with a minimal cost function. Simulations utilising these values (Fig. A) are indistinguishable from each other demonstrating that fitting Model A to data results in many distinct parameter sets that describe the data equally well.

Fig. B presents the sensitivity scores (see the main paper (Methods) for a description of the technique used) corresponding to the effect that variation in the parameters obtained from fitting the model to

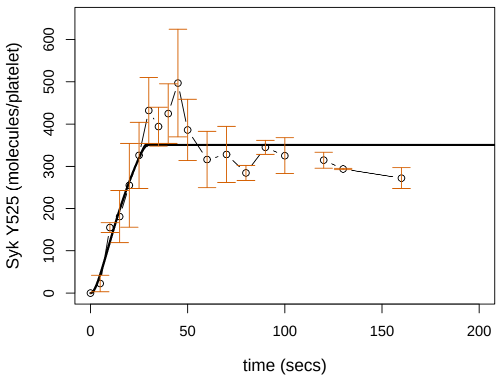

Figure A. Model A simulations (−) compared to experimental data (− ∘ −). Simulations utilise five optimal sets of parameter values obtained from the parameter fitting process (see Table C) and demonstrate that utilising these sets results in simulations that are indistinguishable, they all describe the data equally well. Model A is unable to describe the early peak in phosphorylation displayed in the experimental observations.

experimental data have on the model profiles representing Syk activity (both its steady state and the time to reach that steady state). These scores demonstrate that Model A's steady state is determined by the rate of Syk phosphorylation (p_1) and its reverse process (γ_1), the ratio of which is constant for the five 'best' fits ($p_1/\gamma_1 = 0.145$, see Table C). The time to reach the steady state is mainly sensitive to variation in the rate that the receptor complex is phosphorylated (k_2). Utilising equations (S1) and (S2) we can see that at steady state, in the regime where $l_I >> g_I > s_I$, all Syk molecules are bound to the receptor ($s = 0$) and the number of inactive and active receptors are given by

$$G_0^b = \frac{\gamma_1}{\gamma_1 + p_1} s_I \qquad \text{and} \qquad G_1^b = \frac{p_1}{\gamma_1 + p_1} s_I. \tag{S3}$$

S.2 Model B

Model B extends Model A to incorporate the hypothesis that Syk activity is regulated via a negative feedback pathway initiated through a newly introduced Syk phosphorylation site (Y323). Phosphorylation on Y323 is thought to allow the cytosolic protein c-Cbl to bind to Syk where it facilitates ubiquitination and subsequent binding of the cytosolic protein TULA-2 that is a phosphatase thought able to desphosphorylate Syk Y525. A network diagram representing the events incorporated in Model B is shown in Fig. 3 while a summary of the variables and the newly introduced dimensional parameters is presented in Tables D and E, respectively. The remaining parameters are the same as those for Model A (Table B). Incorporating these reactions into Model A leaves equations (S1a)-(S1c), and (S1g) unchanged.

Model A's variables G_0^b and G_1^b are modified to the form $G_{i,j}^k$ which represents Syk (bound to the receptor complex) in its various states. Subscripts i and j indicate phosphorylation on sites Y323 and Y525 respectively and superscript k denotes the sequential events of Syk binding to the receptor complex

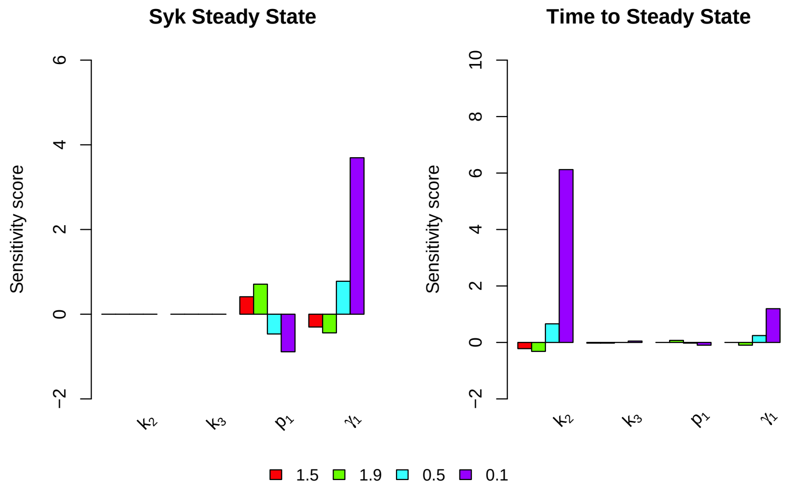

Figure B. Sensitivity of Model A to variation in the model's fitted parameter values. Sensitivity scores are shown for the effect of parameter variation on variable G_1^b (representing Syk activity). Parameters here are varied ± 50% and 90%.

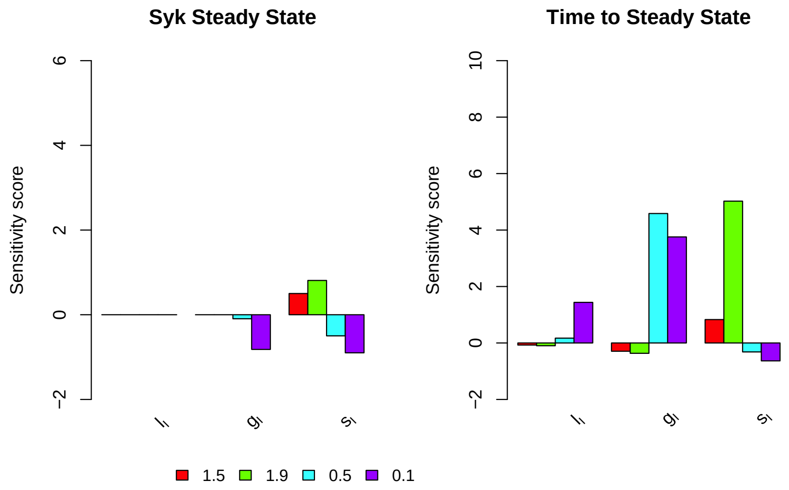

Figure C. Sensitivity of Model A to variation in the model's initial conditions. Sensitivity scores are shown for the effect of parameter variation on variable $\mathbf{G_1^b}$ (representing Syk activity). Parameters here are varied ± 50% and 90%.

(b), c-Cbl binding (c), Syk's ubiquitination (u) and TULA-2 binding to Syk (r). In Model B the equations (S1d) and (S1e) are therefore replaced by the following ten differential equations where terms unchanged from those in Model A are shown in black and those newly introduced in blue

$$\frac{dG^b_{0,0}}{dt} = \frac{k_3}{V_pA_v}\,G_p\,s - p_2\,G^b_{0,0} + p_{-2}\,G^b_{1,0} - p_1\,G^b_{0,0}, \tag{S4a}$$

$$\frac{dG^b_{1,0}}{dt} = p_2G^b_{0,0} - p_{-2}\,G^b_{1,0} - \frac{p_3}{V_pA_v}G^b_{1,0}\,c + p_{-3}\,G^c_{1,0} - p_1\,G^b_{1,0}, \tag{S4b}$$

$$\frac{dG^c_{1,0}}{dt} = \frac{p_3}{V_pA_v}G^b_{1,0}\,c - p_{-3}G^c_{1,0} - p_1\,G^c_{1,0} - p_4\,G^c_{1,0} + p_{-4}\,G^u_{1,0}, \tag{S4c}$$

$$\frac{dG^u_{1,0}}{dt} = p_4\,G^c_{1,0} - p_{-4}\,G^u_{1,0} - p_1\,G^u_{1,0} - \frac{p_5}{V_pA_v}\,G^u_{1,0}\,r + p_{-5}\,G^r_{1,0}, \tag{S4d}$$

$$\frac{dG^r_{1,0}}{dt} = \frac{p_5}{V_pA_v}\,G^u_{1,0}\,r - p_{-5}\,G^r_{1,0} - p_1\,G^r_{1,0} + \gamma_1\,G^r_{1,1}, \tag{S4e}$$

$$\frac{dG^b_{0,1}}{dt} = p_1\,G^b_{0,0} - p_2G^b_{0,1} + p_{-2}G^b_{1,1}, \tag{S4f}$$

$$\frac{dG^b_{1,1}}{dt} = p_1\,G^b_{1,0} + p_2G^b_{0,1} - p_{-2}G^b_{1,1} - \frac{p_3}{V_pA_v}\,G^b_{1,1}\,c + p_{-3}\,G^c_{1,1}, \tag{S4g}$$

$$\frac{dG^c_{1,1}}{dt} = p_1\,G^c_{1,0} + \frac{p_3}{V_pA_v}G^b_{1,1}\,c - p_{-3}\,G^c_{1,1} - p_4\,G^c_{1,1} + p_{-4}\,G^u_{1,1}, \tag{S4h}$$

$$\frac{dG^u_{1,1}}{dt} = p_1\,G^u_{1,0} + p_4G^c_{1,1} - p_{-4}\,G^u_{1,1} - \frac{p_5}{V_pA_v}G^u_{1,1}\,r + p_{-5}\,G^r_{1,1}, \tag{S4i}$$

$$\frac{dG^r_{1,1}}{dt} = p_1\,G^r_{1,0} + \frac{p_5}{V_pA_v}G^u_{1,1}\,r - p_{-5}\,G^r_{1,1} - \gamma_1\,G^r_{1,1}. \tag{S4j}$$

We introduce two new equations to describte cytosolic c-Cbl and TULA-2

$$\frac{dc}{dt} = -\frac{p_3}{V_pA_v}\,G^b_{1,0}c + p_{-3}\,G^c_{1,0} - \frac{p_3}{V_pA_v}\,G^b_{1,1}c + p_{-3}\,G^c_{1,1}, \tag{S4k}$$

$$\frac{dr}{dt} = -\frac{p_5}{V_pA_v}\,G^u_{1,0}q + p_{-5}\,G^r_{1,1} - \frac{p_5}{V_pA_v}\,G^u_{1,1}r + p_{-5}\,G^r_{1,0}. \tag{S4l}$$

The initial conditions are set so that

$$l = l_I, \quad g = g_I, \quad G = 0, \quad G_p = 0, \quad G^k_{i,j} = 0, \quad s = s_I, \quad c = c_I, \quad r = r_I, \tag{S5}$$

where we obtain the number of c-Cbl molecules per platelet experimentally and the number of TULA-2 molecules per platelet is given by [2], so that $c_I = 2581$ molecules and $s_I = 7800$ molecules. The newly introduced variables and parameters are described in Tables D and E respectively. In Model A the variable G^b_1 was compared to experimental data of Syk Y525 phosphorylation. In Model B experimental data is compared with G_{Y525} where

$$G_{Y525} = G^b_{0,1} + G^b_{1,1} + G^c_{1,1} + G^u_{1,1} + G^r_{1,1}, \tag{S6}$$

and data describing phosphorylation of Syk on Y323 is compared to G_{Y323} where

$$G_{Y323} = G^b_{1,0} + G^c_{1,0} + G^u_{1,0} + G^r_{1,0} + G^b_{1,1} + G^c_{1,1} + G^u_{1,1} + G^r_{1,1}. \tag{S7}$$

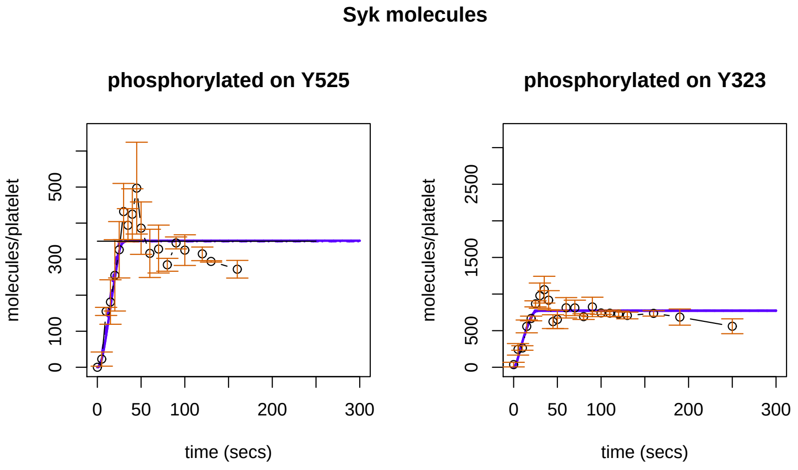

Figure D. Model B simulations (−) compared to experimental data (− ○ −) describing phosphorylation on Y525 and Y323. Simulations utilise the five optimal set of parameter values listed in Table G and demonstrate that utilising these sets results in simulations that are indistinguishable.

The expressions

$$l_0 - l = G + G_p + G^b_{0,0} + G^b_{1,0} + G^c_{1,0} + G^u_{1,0} + G^r_{1,0} + G^b_{0,1} + G^b_{1,1} + G^c_{1,1} + G^u_{1,1} + G^r_{1,1}, \quad (S8)$$

$$g_0 - g = G + G_p + G^b_{0,0} + G^b_{1,0} + G^c_{1,0} + G^u_{1,0} + G^r_{1,0} + G^b_{0,1} + G^b_{1,1} + G^c_{1,1} + G^u_{1,1} + G^r_{1,1}, \quad (S9)$$

follow from (S1a)-(S1c), (S1g) and (S4) and therefore

$$g - l = \text{constant}. \quad (S10)$$

Conservation of Syk, c-Cbl and TULA-2 given by

$$s_I - s = G^b_{0,0} + G^b_{1,0} + G^c_{1,0} + G^u_{1,0} + G^r_{1,0} + G^b_{0,1} + G^b_{1,1} + G^c_{1,1} + G^u_{1,1} + G^r_{1,1}, \quad (S11)$$

$$c_I - c = G^c_{1,0} + G^u_{1,0} + G^r_{1,0} + G^c_{1,1} + G^u_{1,1} + G^r_{1,1}, \quad (S12)$$

$$r_I - r = G^r_{1,0} + G^r_{1,1}. \quad (S13)$$

S.3 Model B solutions

Model B has eight newly introduced parameters (listed in Table E). Parameters p_4 and p_{-4} describe ubiquitination and its reverse process. [3] provide an estimate for ubiquitination (1.67 s^{-1}) that is of a similar order to estimates for phosphorylation and this leads us to set $p_3 = p_{-3} = 1 \times 10^{-2} - 10^3$ s^{-1}. The remaining parameters (p_2, p_{-2}, p_3, p_{-3}, p_5, p_{-5}) describe phosphorylation, binding or their reverse processes and we therefore follow the arguments described in Section S.1.1 in setting the constraints that their values can take during the fitting process.

The number of parameters that require estimation has increased from four, in Model A, to twelve in Model B. As in Model A we fixed the parameters k_1, k_{-1}, V_p, V_e to the values described in Table B and

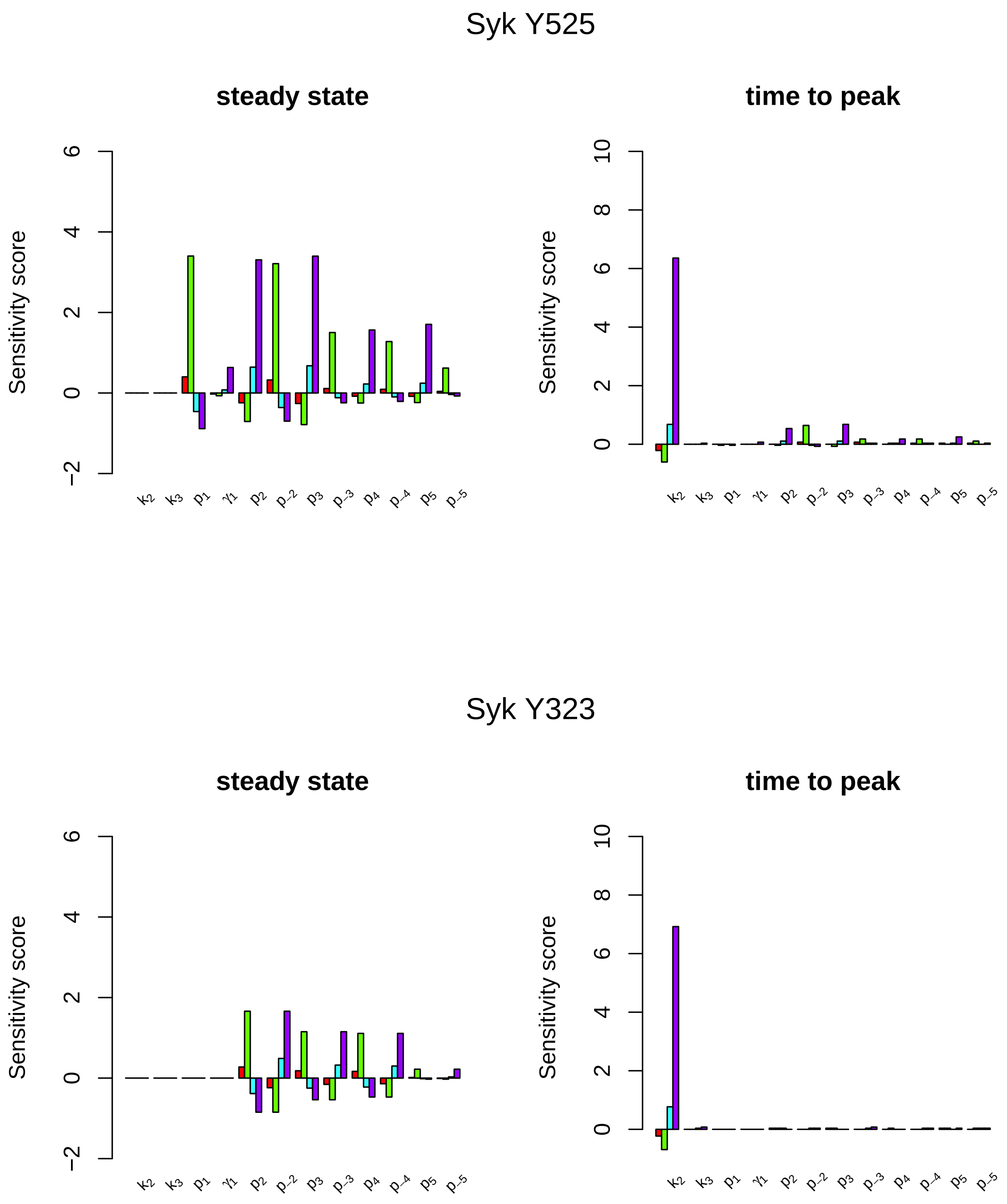

Figure E. Sensitivity of Model B to variation in the model's fitted parameter values. Parameters here are varied ± 50% and 90%.

then infer the remaining parameters from data. The five 'best' parameter sets obtained from fitting model B to experimental data describing Syk 525 phosphorylation and then simultaneously fitting the model to data describing Syk phosphorylation on Y525 and Y323 are shown in Tables F and G respectively. Simulations utilising these values are indistinguishable from each other demonstrating that the fitting process has converged (see Fig. D for simulations that utilise the five optimal sets of parameters obtained from fitting Model B to observations of Y525 and Y323).

Model B fitted to data describing the activatory phosphorylation site (Y525) is able to describe experimental observations of Y525 but model predictions for the regulatory phosphorylation site (Y323) settle to a steady state that is approximately twice that described by data (see Fig. 4a and 6b). Model B fitted simultaneously to experimental data describing both phosphorylation sites (see Fig. 4c and 4d) is able to describe the steady states that experimental observations settle to but is unable to describe the early transient peaks displayed in the data.

Fig.E presents the sensitivity scores corresponding to the effect that variation in the parameters obtained from fitting the model to experimental data, describing phosphorylation on Y525 and Y323, have on the model profiles representing both phosphorylation sites (their steady states and the time to reach that steady state). These scores demonstrate that the time to reach the steady state in Syk Y525 (and indeed in Y323) is, like Model A, mainly sensitivie to variation in the rate that the receptor complex is phosphorylated (k_2). Utilising equations (S4) and (S13) we can see that at steady state, in the regime where $l_I >> g_I > s_I$, all Syk molecules are bound to the receptor ($s = 0$) and the number of active receptors are given by

$$G_{Y525} = S_I - \frac{\gamma_1}{p_1} G^b_{1,1} \tag{S14}$$

where $G^b_{1,1}$ (the proportion of active Syk with TULA-2 bound) reflects Syk activity participating in its own regulation. The steady states of Syk Y525 is now sensitive to variation in the rate of Syk phosphorylation and the parameters that comprise the regulatory pathway.

S.4 Model C

In formulating Model C we explored the following biological hypothesis:

H1 : TULA-2 (once bound to the receptor complex) is able to dephosphorylate not only the Syk molecule to which it is associated but any nearby Syk molecule (at rate γ_2),

H2 : Syk activity (phosphorylation on Y525) increases the rate of phosphorylation of Y323 (denoted by q_2),

H3 : H1 and H2 incorporated simultaneously.

A network diagram representing Model C is shown in Fig. 3 (lower panel). The newly introduced parameters (q_2, γ_2) are presented in Table H. All other parameters and all variables are the same as those for Model B (see Tables B, E and D). Incorporating the hypotheses into the equations for Model B leaves equations (S1a)-(S1c), (S1g), (S4e) and (S4j-S4l) unchanged. Incorporating H1, the ability of TULA-2 to dephosphorylate nearby receptors (rate γ_2), requires additional terms in equations (S4a-S4d, S4f-S4i) and incorporating H2 (the increase in the rate that Y323 is phosphorylated (by q_2) results in the modifications to equations (S4f) and (S4g). These equations (with the modifications for H1 and

H2 depicted in blue and green respectively) now read

$$\frac{dG^b_{0,0}}{dt} = \frac{k_3}{V_pA_v} G_p\, s - p_2\, G^b_{0,0} + p_{-2}\, G^b_{1,0} - p_1\, G^b_{0,0} + \gamma_2 \left(G^r_{1,1} + G^r_{1,0}\right) G^b_{0,1}, \tag{S15a}$$

$$\frac{dG^b_{1,0}}{dt} = p_2 G^b_{0,0} - p_{-2}\, G^b_{1,0} - \frac{p_3}{V_pA_v} G^b_{1,0}\, c + p_{-3}\, G^c_{1,0} - p_1\, G^b_{1,0} + \gamma_2 \left(G^r_{1,1} + G^r_{1,0}\right) G^b_{1,1}, \tag{S15b}$$

$$\frac{dG^c_{1,0}}{dt} = \frac{p_3}{V_pA_v} G^b_{1,0}\, c - p_{-3} G^c_{1,0} - p_1\, G^c_{1,0} - p_4\, G^c_{1,0} + p_{-4}\, G^u_{1,0} + \gamma_2 \left(G^r_{1,1} + G^r_{1,0}\right) G^c_{1,1}, \tag{S15c}$$

$$\frac{dG^u_{1,0}}{dt} = p_4\, G^c_{1,0} - p_{-4}\, G^u_{1,0} - p_1\, G^u_{1,0} - \frac{p_5}{V_pA_v} G^u_{1,0}\, r + p_{-5}\, G^r_{1,0} + \gamma_2 \left(G^r_{1,1} + G^r_{1,0}\right) G^u_{1,1}, \tag{S15d}$$

$$\frac{dG^b_{0,1}}{dt} = p_1\, G^b_{0,0} - q_2\, p_2 G^b_{0,1} + p_{-2} G^b_{1,1} - \gamma_2 \left(G^r_{1,1} + G^r_{1,0}\right) G^b_{0,1}, \tag{S15e}$$

$$\frac{dG^b_{1,1}}{dt} = p_1\, G^b_{1,0} + q_2\, p_2 G^b_{0,1} - p_{-2} G^b_{1,1} - \frac{p_3}{V_pA_v} G^b_{1,1}\, c + p_{-3}\, G^c_{1,1} - \gamma_2 \left(G^r_{1,1} + G^r_{1,0}\right) G^b_{1,1}, \tag{S15f}$$

$$\frac{dG^c_{1,1}}{dt} = p_1\, G^c_{1,0} + \frac{p_3}{V_pA_v} G^b_{1,1}\, c - p_{-3}\, G^c_{1,1} - p_4\, G^c_{1,1} + p_{-4}\, G^u_{1,1} - \gamma_2 \left(G^r_{1,1} + G^r_{1,0}\right) G^c_{1,1}, \tag{S15g}$$

$$\frac{dG^u_{1,1}}{dt} = p_1\, G^u_{1,0} + p_4 G^c_{1,1} - p_{-4}\, G^u_{1,1} - \frac{p_5}{V_pA_v} G^u_{1,1}\, r + p_{-5}\, G^r_{1,1} - \gamma_2 \left(G^r_{1,1} + G^r_{1,0}\right) G^u_{1,1}, \tag{S15h}$$

The initial conditions and conservation laws for Model C are as described for Model B (equations (S5)-(S13)).

By setting $q_2 = 1$ and $\gamma_2 = 0$ we recover Model B. By setting $q_2 = 1$ we obtain Model C (H1) and setting $\gamma_2 = 0$ to obtain Model C (H2).

S.5 Model C solutions

Model C has two newly introduced parameters (listed in Table H). The parameter γ_2 describes dephosphorylation of Y525 but, unlike the parameter (γ_1) introduced in Model B, is second order. As there are no estimates for the second order form (units molecules^{-1} s^{-1}) in literature we set a wide range that the parameter is allowed to take in the parameter fitting process (1.0×10^{-3} to 1×10^4 molecules s^{-1}). The parameter q_2 is dimensionless, it increases the rate that of Syk phosphorylation p_2 and we therefore allow it to take the range 1.0 to 1×10^4.

We incorporate each hypothesis and fit the model to experimental data. The model is fitted twice, initially to data describing phosphorylation on Y525 and then to data describing phosphorylation on Y525 and Y323. The five 'best' parameter sets obtained from fitting Model C (with H1, H2 and then H3 incorporated) are shown in Tables I and J. Simulations utilising the optimal parameter sets from fitting Model C simultaneously to data describing both phosphorylation sites are shown in Fig 5c and 5d. These demonstrate that Model C with hypothesis H3 is best able to accurately describe Syk phosphorylation on Y525 and Y323. Model C with H1 or H2 incorporated fail to capture the full dynamics displayed in the data. If experimental data is restricted to one phosphorylation site ($Y525$) then Model C, like Model B, is able to describe Syk Y525 phosphorylation but predictions for Y323 phosphorylation are inaccurate. When fitting Model C, H3 a sample size of N=10000 was used, this larger sample size being required to fit the more complicated model. Fig. F demonstrates that simulations utilising the five best parameter sets (Table J, $H3, 1-5$) are indistinguishable; the parameter fitting process has converged to parameter

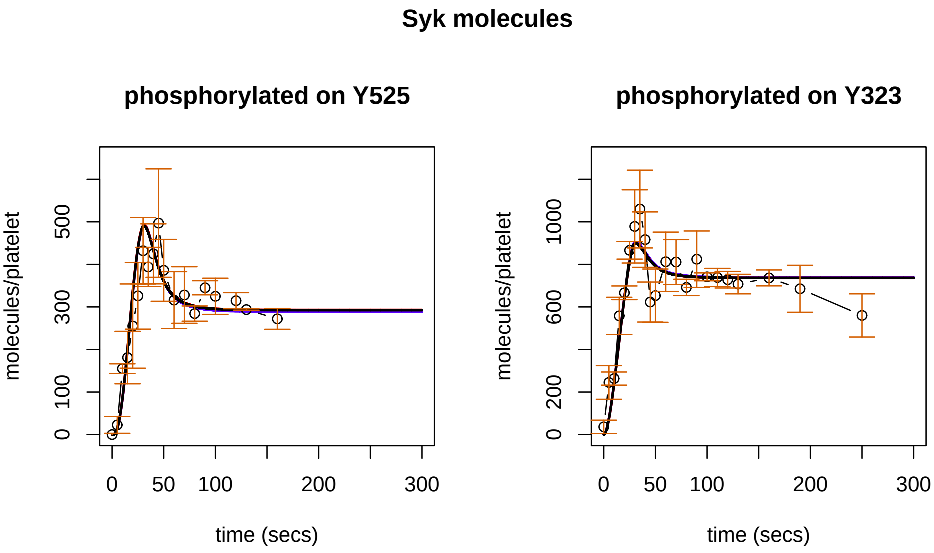

Figure F. Model C simulations $(-)$ compared to experimental data $(-\circ-)$ describing phosphorylation on Y525 and Y323. Simulations utilise the five optimal set of parameter values listed in Table J $(\mathrm{H}3, 1-5)$ and demonstrate that utilising these sets results in simulations that are indistinguishable.

sets that describe the data equally well.

Fig. G,H,I and J presents the sensitivity scores corresponding to the effect that variation in the parameters and initial conditions of Model C (H$-$3) have on the model profiles representing phosphorylation on Syk Y525 and Y323 (both their steady states and the time to reach that steady state). These scores demonstrate that the time to reach both steady states is predominantly influenced by the rate that the receptor complex is phosphorylated (k_2) with the time to reach the steady state of Y323 also being strongly influenced by the initial conditions of GPVI and Syk. Of the paremeters that were held fixed during the parameter fitting process the volume has the most influence on the model outputs. The steady state in Syk Y525 phosphorylation is influenced by all parameters that comprise the regulatory pathway and the levels of the regulatory proteins (c-Cbl and TULA-2). Utilising Model C equations (S15) and (S13) we can see that at steady state, in the regime where $l_I >> g_I > s_I$, all Syk molecules are bound to the receptor ($s = 0$) and the number of active receptors are given by

$$G_{Y525} = S_I - \frac{\gamma_1}{p_1} G^b_{1,1} - \frac{\gamma_2}{p_1} \left(G^b_{1,0} + G^b_{1,1}\right) \left(G^b_{0,1} + G^b_{1,1} + G^c_{1,1} + G^u_{1,1}\right). \quad \text{(S16)}$$

where $(G^b_{1,0} + G^b_{1,1})$ represents TULA-2 bound to the receptor complex and $(G^b_{0,1} + G^b_{1,1} + G^c_{1,1} + G^u_{1,1})$ represents the portion of Syk phosphorylated on Y525 but without TULA-2 bound.

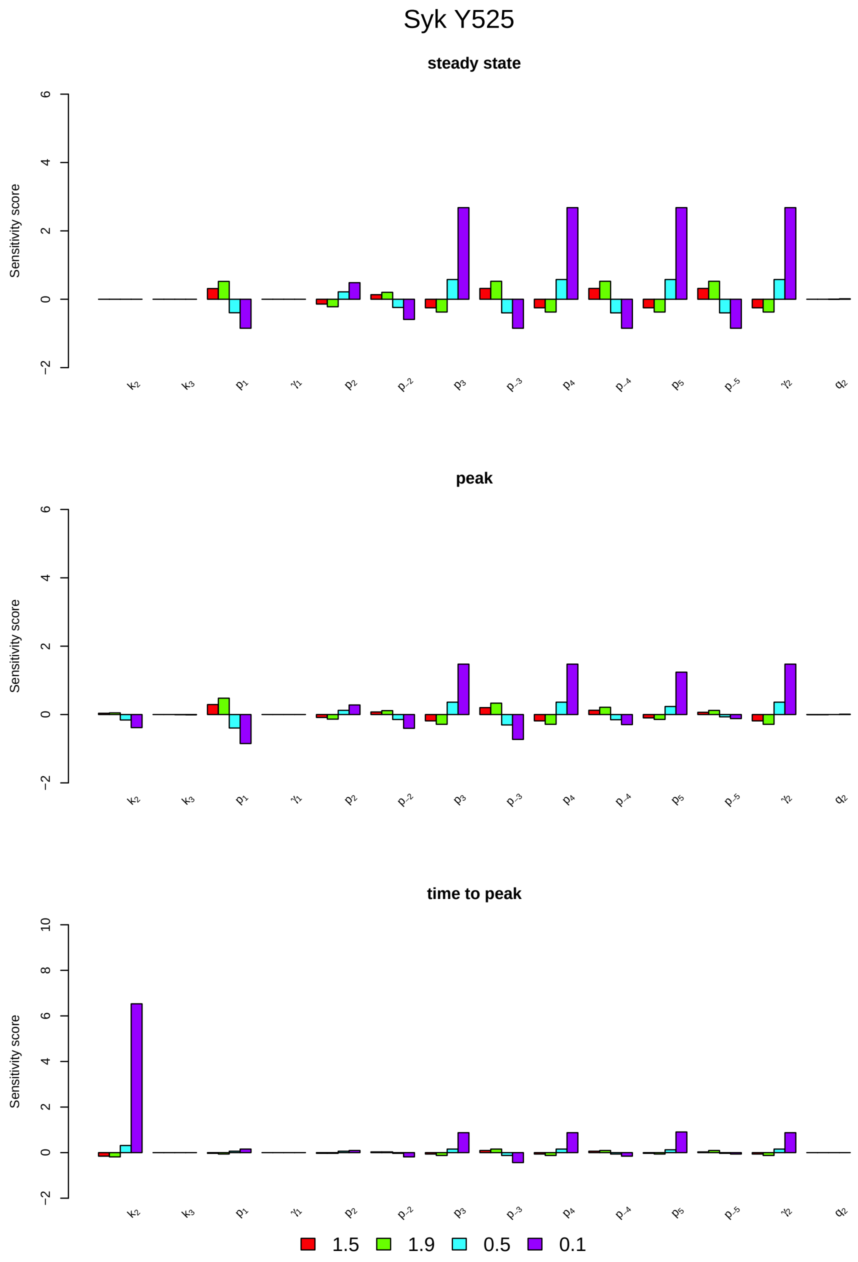

Figure G. Sensitivity of Model C to variation in model parameters that were inferred from data. Parameters here are varied ± 50% and 90%. The effect of variation on markers of model output (steady state, maximum peak and time-to-reach peak) for Syk phosphorylation on Y525 are shown.

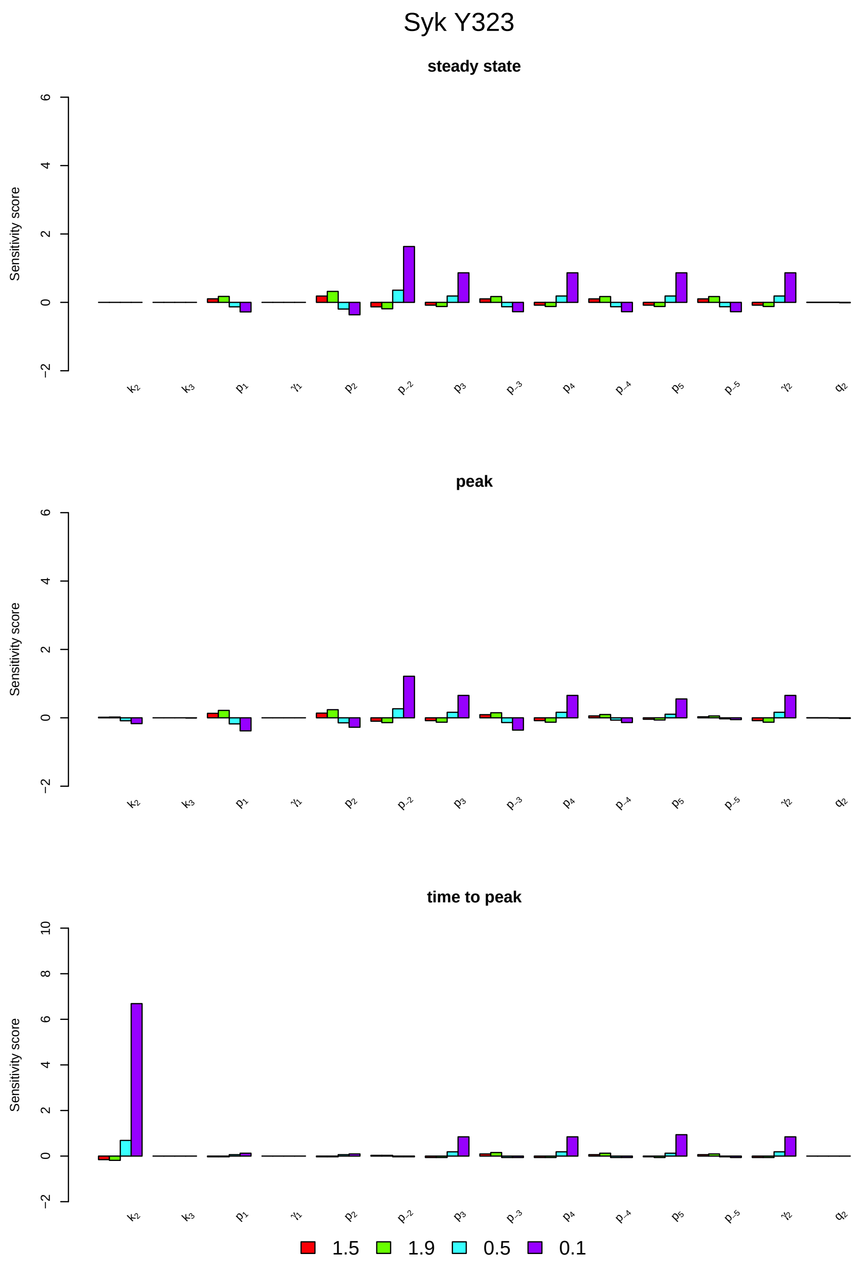

Figure H. Sensitivity of Model C to variation in model parameters that were inferred from data. Parameters here are varied ± 50% and 90%. The effect of variation on markers of model output (steady state, maximum peak and time-to-reach peak) for Syk phosphorylation on Y323 are shown.

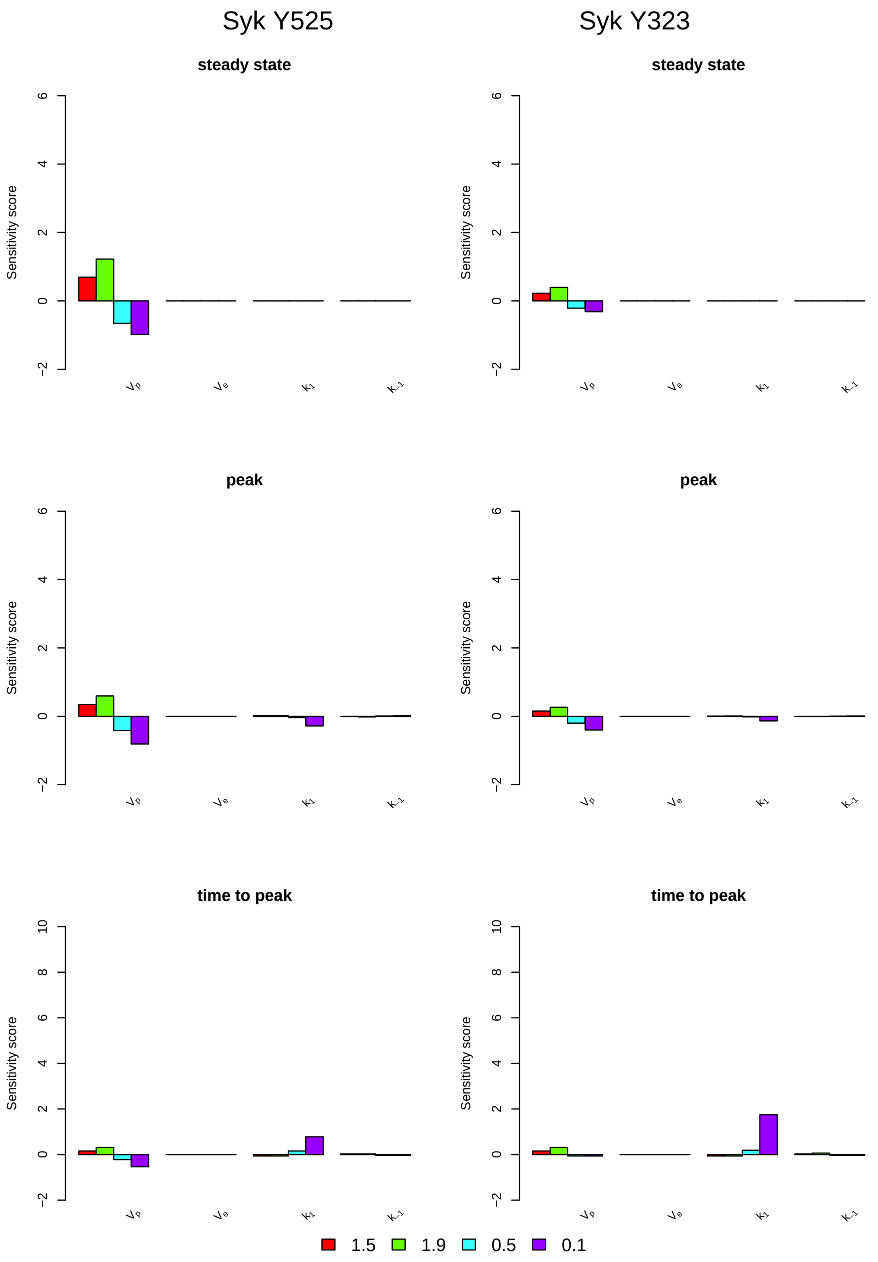

Figure I. Sensitivity of Model C to variation in model parameters that were held fixed during the parameter fitting process. Parameters here are varied ± 50% and 90%. The effect of variation on markers of model output (steady state, maximum peak and time-to-reach peak) for Syk phosphorylation on Y525 and Y323 are shown.

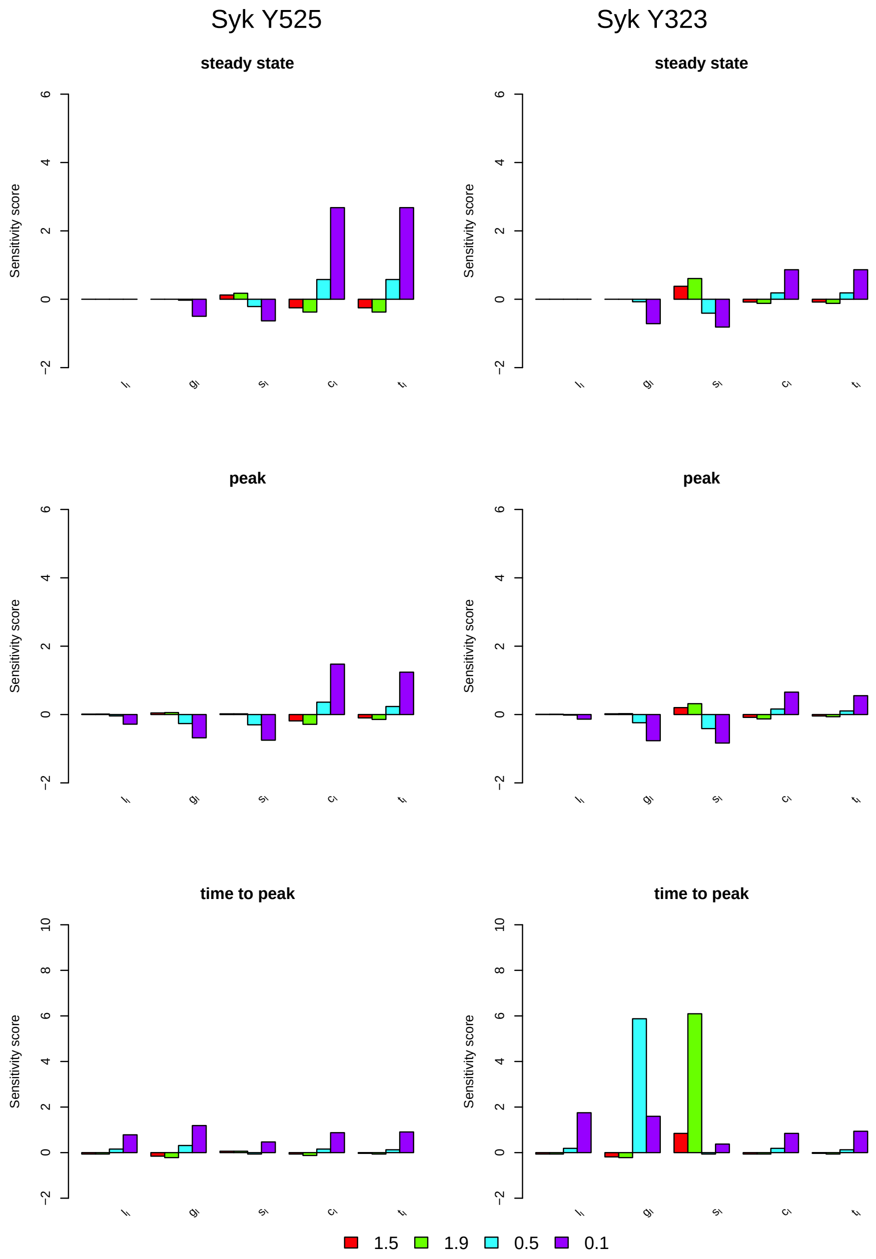

Figure J. Sensitivity analysis of Model C to variation in the initial conditions. Parameters here are varied ± 50% and 90%. The effect of variation on markers of model output (steady state, maximum peak and time-to-reach peak) for Syk phosphorylation on Y525 and Y323 are shown.

Variable	Description	Units	Initial condition	Source
l	Ligand	moles m^{-3}	3×10^{-2}	estimated
g	GPVI	molecules	5000	this study
s	Syk, cytosolic	molecules	2763	this study
G	Ligand bound to GPVI	molecules	0	
G^p	phosphorylated receptor	molecules	0	
G_0^b	Syk bound to receptor	molecules	0	
G_1^b	Syk phosphorylated on Y525	molecules	0	

Table A. Model A variables.

Parameter	Description	Units	Values	Source
k_1	rate of ligand binding	m^3 moles^{-1} s^{-1}	8	[10]
k_{-1}	ligand dissociation constant	s^{-1}	3.02×10^{-2}	[10]
V_e	extracellular volume per cell	m^3	3.3×10^{-9}	this study
V_p	platelet volume	m^3	7.4×10^{-18}	this study
k_2	rate of ITAM phosphorylation	s^{-1}	1.0×10^{-2} to 1×10^3	[3,5,11]
k_3	rate Syk binds to receptor bundle	s^{-1}	1.0×10^1 to 1×10^6	[5–8,11,12]
p_1	rate Syk phosphorylates	s^{-1}	1.0×10^{-2} to 1×10^3	[3,5,11]
γ_1	rate Syk dephosphorylates	s^{-1}	1.0×10^{-3} to 1×10^3	[5,7,8,11,12]

Table B. Model A parameters. Section S.1.1 gives details of how we the obtained the parameter values described in the top four rows Section S.1.2 describes how we obtain the fitting constraints.

parameter set	SSE	k_2	k_3	p_1	γ_1	p_1/γ_1
1	$6.65e^4$	$3.87e^{-2}$	$9.55e^5$	$5.13e^{-1}$	3.53	$1.45e^{-1}$
2	$6.65e^4$	$3.87e^{-2}$	$9.44e^5$	$5.68e^{-1}$	3.91	$1.45e^{-1}$
3	$6.65e^4$	$3.85e^{-2}$	$3.05e^5$	$7.26e^{-1}$	5.00	$1.45e^{-1}$
4	$6.65e^4$	$3.87e^{-2}$	$3.46e^5$	$5.38e^{-1}$	3.70	$1.45e^{-1}$
5	$6.65e^4$	$3.86e^{-2}$	$2.60e^5$	$6.77e^{-1}$	4.66	$1.45e^{-1}$

Table C. Model A's estimated parameter values: a range of the 'best' (lowest SSE) parameter sets obtained when fitting Model A output to experimental data.

Variable	Description	Units	Initial condition	Source
l	Ligand	moles m^{-3}	3×10^{-2}	estimated
g	GPVI	molecules	5000	this study
s	Syk, cytosolic	molecules	2763	this study
G	Ligand bound to GPVI	molecules	0	
G^p	phosphorylated receptor	molecules	0	
$G_{i,j}^k$	Syk bound to receptor	molecules	0	
r	TULA-2	molecules	7800	[2]
c	c-Cbl	molecules	2581	this study

Table D. Model B and C's variables. The top five variables are common to those of Model A. $G_{i,j}^k$ represents eight variables where i and j indicate phosphorylation on Y323 and Y525 respectively (0, unphosphorylated; 1, phosphorylated) and k denotes the sequential processes of (b), binding of Syk; (c), binding of c-Cbl; (u), ubiquitination and (r), binding of TULA-2.

Parameter	Description	Units	Fitting constraints	Source
p_2	Syk phosphorylation (Y323)	s^{-1}	1.0×10^{-2} to 1×10^{3}	[3,5,11]
p_{-2}	Syk Y323 regulation	s^{-1}	1.0×10^{-3} to 1×10^{3}	[5,7,8,11,12]
p_3	c-Cbl binding	m^3 moles^{-1} s^{-1}	1.0×10^{1} to 1×10^{6}	[5–8,11,12]
p_{-3}	c-Cbl dissociation	s^{-1}	1.0×10^{-3} to 1×10^{3}	[5,7,8,11,12]
p_4	ubiquitination	s^{-1}	1.0×10^{-2} to 1×10^{3}	[3]
p_{-4}	deubiquitination	s^{-1}	1.0×10^{-3} to 1×10^{3}	[3]
p_5	TULA-2 binding	m^3 moles^{-1} s^{-1}	1.0×10^{1} to 1×10^{6}	[5–8,11,12]
p_{-5}	TULA-2 dissociation	s^{-1}	1.0×10^{-3} to 1×10^{3}	[5,7,8,11,12]

Table E. Model B's newly introduced parameters. Their definitions, units and fitting constraints (upper and lower bound) utilized by the parameter fitting process (see the main paper (Methods) for a description of the parameter estimation technique and Section S.3 for details of how parameter ranges were set). Model B's remaining parameters are common to Model A (see Table B).

parameter set	SSE	p_1	γ_1	p_2	p_3	p_4	p_5
1	$2.61e^{4}$	$5.85e^{-2}$	$7.25e^{-1}$	$1.04e^{-1}$	$5.89e^{5}$	$9.82e^{-1}$	$9.29e^{3}$
2	$2.61e^{4}$	$5.47e^{-2}$	$1.54e^{1}$	$8.82e^{-2}$	$1.53e^{5}$	$8.06e^{1}$	$2.42e^{2}$
3	$2.61e^{4}$	$5.76e^{-2}$	$1.02e^{0}$	$8.48e^{-2}$	$5.28e^{4}$	$3.05e^{2}$	$1.80e^{4}$
4	$2.62e^{4}$	$5.13e^{-2}$	$7.13e^{-1}$	$9.04e^{-2}$	$1.35e^{5}$	$7.07e^{-1}$	$2.75e^{4}$
5	$2.62e^{4}$	$6.11e^{-2}$	$1.06e^{0}$	$8.82e^{-2}$	$9.99e^{3}$	$2.62e^{2}$	$2.85e^{5}$
parameter set	SSE	k_2	k_3	p_{-2}	p_{-3}	p_{-4}	p_{-5}
1	$2.61e^{4}$	$2.32e^{-2}$	$7.74e^{5}$	$2.17e^{-2}$	$2.19e^{-1}$	$3.36e^{0}$	$1.69e^{-1}$
2	$2.61e^{4}$	$2.39e^{-2}$	$2.81e^{4}$	$1.00e^{-2}$	$1.35e^{-1}$	$2.35e^{0}$	$2.57e^{-1}$
3	$2.61e^{4}$	$2.37e^{-2}$	$3.36e^{4}$	$1.00e^{-2}$	$1.04e^{-1}$	$2.19e^{1}$	$5.27e^{0}$
4	$2.62e^{4}$	$2.36e^{-2}$	$5.24e^{4}$	$1.00e^{-2}$	$2.07e^{-1}$	$3.08e^{0}$	$8.98e^{-2}$
5	$2.62e^{4}$	$2.37e^{-2}$	$1.27e^{5}$	$1.00e^{-2}$	$3.84e^{-2}$	$8.47e^{1}$	$2.45e^{1}$

Table F. Results of parameter fitting Model B to experimental observations of Syk Y525 phosphorylation. Parameter sets the five best results from a sample of $N = 1000$ fits.

parameter set	SSE	p_1	γ_1	p_2	p_3	p_4	p_5
1	$3.56e^{5}$	$1.83e^{-1}$	$9.96e^{2}$	$2.62e^{1}$	$3.39e^{4}$	$8.20e^{2}$	$2.11e^{4}$
2	$3.57e^{5}$	$6.76e^{-2}$	$1.16e^{2}$	$3.05e^{0}$	$3.98e^{4}$	$1.84e^{2}$	$4.42e^{3}$
3	$3.58e^{5}$	$5.42e^{-2}$	$2.12e^{1}$	$3.33e^{1}$	$2.62e^{5}$	$8.81e^{1}$	$3.03e^{3}$
4	$3.58e^{5}$	$8.15e^{-2}$	$1.36e^{2}$	$1.24e^{0}$	$9.21e^{4}$	$8.00e^{1}$	$7.75e^{4}$
5	$3.59e^{5}$	$7.01e^{-2}$	$9.98e^{2}$	$8.80e^{0}$	$9.00e^{3}$	$8.21e^{2}$	$7.80e^{3}$
parameter set	SSE	k_2	k_3	p_{-2}	p_{-3}	p_{-4}	p_{-5}
1	$3.56e^{5}$	$4.57e^{-2}$	$5.05e^{5}$	$2.28e^{2}$	$7.24e^{1}$	$8.74e^{1}$	$4.25e^{2}$
2	$3.57e^{5}$	$4.66e^{-2}$	$9.38e^{4}$	$3.52e^{1}$	$1.69e^{1}$	$1.14e^{2}$	$1.69e^{1}$
3	$3.58e^{5}$	$4.49e^{-2}$	$3.46e^{6}$	$9.67e^{2}$	$3.64e^{1}$	$7.91e^{1}$	$4.39e^{0}$
4	$3.58e^{5}$	$5.11e^{-2}$	$1.59e^{4}$	$4.84e^{0}$	$1.41e^{2}$	$3.46e^{2}$	$1.14e^{2}$
5	$3.59e^{5}$	$5.23e^{-2}$	$9.23e^{3}$	$4.71e^{1}$	$2.77e^{1}$	$1.60e^{2}$	$1.20e^{2}$

Table G. Optimal parameter sets obtained from fitting Model B to experimental observations of Syk Y525 and Y323 phosphorylation. Parameter sets are the optimal results from a sample of $N = 1000$ fits.

Parameter	Description	Units	Fitting constraints
γ_2	dephosphorylation of Y525	molecules^{-1} s^{-1}	1.0×10^{-3} to 1×10^4
q_2	increase in Syk Y323	dimensionless	1.0×10^{-8} to 1×10^4

Table H. Model C's newly introduced parameters. Their definitions, units and fitting constraints (upper and lower bound) utilized by the parameter fitting process (see the main paper (Methods) for a description of the parameter estimation technique and Section S.5 for details of how parameter ranges were set). Model C's remaining parameters are common to Model A and B (see Tables B and E respectively).

parameter set	SSE	p_1	γ_1	p_2	p_3	p_4	p_5	γ_2
$H1,1$	$2.81e^{4}$	$5.12e^{-1}$	$7.75e^{2}$	$2.19e^{2}$	$8.00e^{2}$	$5.04e^{1}$	$4.21e^{3}$	$1.57e^{-3}$
$H1,2$	$2.94e^{4}$	$7.85e^{-2}$	$2.93e^{2}$	$7.90e^{-1}$	$4.59e^{2}$	$5.24e^{1}$	$3.72e^{2}$	$1.00e^{-3}$
$H1,3$	$3.16e^{4}$	$1.00e^{-2}$	$1.08e^{-1}$	$1.06e^{-1}$	$9.81e^{1}$	$1.97e^{1}$	$1.00e^{1}$	$2.19e^{-3}$
$H1,4$	$3.17e^{4}$	$1.00e^{-2}$	$4.34e^{2}$	$1.63e^{-2}$	$2.18e^{1}$	$7.89e^{1}$	$1.00e^{1}$	$2.69e^{-2}$
$H1,5$	$3.18e^{4}$	$1.00e^{-2}$	$1.04e^{-2}$	$3.57e^{-2}$	$6.38e^{2}$	$4.21e^{-1}$	$1.01e^{4}$	$6.91e^{-3}$
$H2,1$	$2.52e^{4}$	$8.51e^{-2}$	$1.07e^{1}$	$1.57e^{-2}$	$3.93e^{5}$	$6.08e^{0}$	$2.02e^{3}$	1
$H2,2$	$2.59e^{4}$	$5.80e^{-2}$	$9.54e^{-1}$	$8.15e^{-2}$	$6.88e^{4}$	$5.09e^{1}$	$1.82e^{5}$	1
$H2,3$	$2.65e^{4}$	$5.38e^{-2}$	$1.00e^{3}$	$3.58e^{-2}$	$4.46e^{4}$	$1.60e^{-1}$	$1.34e^{4}$	1
$H2,4$	$2.66e^{4}$	$2.60e^{-1}$	$8.22e^{2}$	$4.70e^{2}$	$1.20e^{3}$	$6.90e^{2}$	$1.16e^{3}$	1
$H2,5$	$2.66e^{4}$	$2.55e^{-1}$	$9.48e^{2}$	$2.52e^{0}$	$6.72e^{2}$	$9.20e^{2}$	$1.79e^{4}$	1
$H3,1$	$2.71e^{4}$	$3.70e^{-1}$	$7.84e^{2}$	$4.20e^{2}$	$7.92e^{2}$	$1.00e^{3}$	$2.95e^{3}$	$1.00e^{-3}$
$H3,2$	$2.77e^{4}$	$1.54e^{-1}$	$7.12e^{2}$	$1.50e^{-1}$	$1.78e^{3}$	$1.20e^{1}$	$2.09e^{3}$	$1.20e^{-3}$
$H3,3$	$2.83e^{4}$	$9.57e^{-2}$	$8.25e^{2}$	$2.64e^{-1}$	$8.48e^{2}$	$4.90e^{0}$	$6.86e^{2}$	$1.00e^{-3}$
$H3,4$	$2.91e^{4}$	$5.87e^{-2}$	$4.45e^{2}$	$1.39e^{-1}$	$2.30e^{2}$	$1.07e^{1}$	$3.85e^{4}$	$3.32e^{-3}$
$H3,5$	$3.02e^{4}$	$4.24e^{-2}$	$1.18e^{1}$	$8.93e^{-1}$	$1.98e^{2}$	$5.92e^{2}$	$1.58e^{3}$	$2.30e^{-3}$

parameter set	SSE	k_2	k_3	p_{-2}	p_{-3}	p_{-4}	p_{-5}	q_2
$H1,1$	$2.81e^{4}$	$2.65e^{-2}$	$1.64e^{4}$	$1.00e^{-2}$	$1.76e^{-2}$	$1.00e^{-2}$	$1.13e^{2}$	0
$H1,2$	$2.94e^{4}$	$2.91e^{-2}$	$4.64e^{3}$	$1.77e^{-1}$	$1.00e^{-2}$	$3.24e^{-2}$	$1.08e^{1}$	0
$H1,3$	$3.16e^{4}$	$8.69e^{2}$	$2.26e^{2}$	$1.00e^{-2}$	$5.66e^{1}$	$1.46e^{-1}$	$1.29e^{-1}$	0
$H1,4$	$3.17e^{4}$	$5.25e^{1}$	$2.28e^{2}$	$1.12e^{-1}$	$4.40e^{-1}$	$3.71e^{1}$	$1.25e^{-1}$	0
$H1,5$	$3.18e^{4}$	$5.87e^{2}$	$2.50e^{2}$	$4.70e^{-2}$	$1.57e^{2}$	$4.22e^{-1}$	$4.18e^{0}$	0
$H2,1$	$2.52e^{4}$	$2.30e^{-2}$	$3.71e^{5}$	$4.01e^{-1}$	$6.20e^{-2}$	$1.08e^{2}$	$2.76e^{-2}$	$3.23e^{2}$
$H2,2$	$2.59e^{4}$	$2.35e^{-2}$	$6.65e^{4}$	$1.00e^{-2}$	$1.72e^{-2}$	$6.89e^{1}$	$1.83e^{1}$	$1.09e^{0}$
$H2,3$	$2.65e^{4}$	$2.38e^{-2}$	$6.90e^{4}$	$1.00e^{-2}$	$3.72e^{-2}$	$7.02e^{-2}$	$3.66e^{2}$	$1.23e^{1}$
$H2,4$	$2.66e^{4}$	$3.44e^{-2}$	$2.60e^{3}$	$1.23e^{-1}$	$3.89e^{2}$	$1.00e^{0}$	$1.24e^{-2}$	$1.00e^{0}$
$H2,5$	$2.66e^{4}$	$3.39e^{-2}$	$2.86e^{3}$	$1.04e^{-2}$	$1.22e^{2}$	$7.97e^{-1}$	$6.55e^{-1}$	$2.67e^{3}$
$H3,1$	$2.71e^{4}$	$3.02e^{-2}$	$3.96e^{3}$	$5.93e^{-1}$	$1.55e^{-1}$	$1.02e^{-2}$	$7.65e^{1}$	$5.04e^{3}$
$H3,2$	$2.77e^{4}$	$3.09e^{-2}$	$5.40e^{3}$	$3.32e^{0}$	$1.46e^{-2}$	$7.25e^{-1}$	$2.18e^{2}$	$3.35e^{1}$
$H3,3$	$2.83e^{4}$	$2.98e^{-2}$	$6.80e^{3}$	$1.28e^{0}$	$6.85e^{-1}$	$1.00e^{-2}$	$2.81e^{1}$	$8.98e^{1}$
$H3,4$	$2.91e^{4}$	$2.86e^{-2}$	$6.31e^{3}$	$1.00e^{-2}$	$1.30e^{-2}$	$6.75e^{1}$	$7.38e^{2}$	$5.16e^{2}$
$H3,5$	$3.02e^{4}$	$2.89e^{-2}$	$9.89e^{3}$	$1.00e^{-2}$	$3.98e^{-1}$	$1.43e^{0}$	$1.03e^{2}$	$8.75e^{2}$

Table I. Results of parameter fitting Model C to experimental observations of Syk Y525 phosphorylation. The results show the five best results utilising the interactions described in hypothesis H1, H2 and H3 utilising a sample of 1000 fits.

parameter set	SSE	p_1	γ_1	p_2	p_3	p_4	p_5	γ_2
$H1,1$	$3.53e^5$	$1.50e^{-2}$	$1.00e^{-002}$	$1.23e^{+001}$	$3.35e^{+003}$	$1.02e^{-001}$	$1.24e^{+002}$	$1.93e^{+001}$
$H1,2$	$3.54e^5$	$1.31e^{-2}$	$1.25e^{+000}$	$7.39e^{-001}$	$4.87e^{+002}$	$1.70e^{-002}$	$1.10e^{+004}$	$1.68e^{+001}$
$H1,3$	$3.55e^5$	$2.66e^{-2}$	$5.76e^{+002}$	$9.60e^{+000}$	$2.78e^{+003}$	$1.01e^{+001}$	$2.65e^{+004}$	$1.33e^{-002}$
$H1,4$	$3.55e^5$	$1.09e^{-2}$	$7.67e^{+002}$	$2.37e^{+000}$	$8.89e^{+003}$	$1.30e^{-001}$	$1.47e^{+001}$	$1.49e^{+000}$
$H1,5$	$3.55e^5$	$1.27e^{-2}$	$2.86e^{+000}$	$1.26e^{+000}$	$3.24e^{+001}$	$1.20e^{-001}$	$3.41e^{+004}$	$8.67e^{+000}$
$H2,1$	$3.57e^5$	$5.22e^{-2}$	$2.18e^2$	$1.01e^1$	$4.70e^4$	$2.15e^1$	$4.29e^3$	1
$H2,2$	$3.60e^5$	$5.64e^{-1}$	$3.35e^2$	$3.37e^0$	$1.02e^4$	$4.53e^2$	$1.20e^5$	1
$H2,3$	$3.60e^5$	$3.93e^{-1}$	$9.94e^2$	$1.39e^1$	$2.52e^4$	$2.99e^2$	$1.46e^4$	1
$H2,4$	$3.60e^5$	$1.31e^{-1}$	$5.42e^2$	$1.10e^1$	$3.98e^5$	$7.83e^2$	$3.35e^3$	1
$H2,5$	$3.61e^5$	$1.56e^{-1}$	$5.49e^2$	$5.15e^0$	$1.01e^4$	$2.72e^2$	$3.18e^4$	1
$H3,1$	$2.43e^5$	$1.44e^{-2}$	$1.00e^{-2}$	$4.48e^0$	$5.67e^2$	$1.73e^{-2}$	$1.00e^1$	$2.50e^{-1}$
$H3,2$	$2.44e^5$	$1.50e^{-2}$	$8.03e^2$	$1.15e^1$	$6.95e^1$	$1.18e^{-2}$	$4.47e^1$	$9.90e^1$
$H3,3$	$2.44e^5$	$1.57e^{-2}$	$5.08e^2$	$2.29e^0$	$9.00e^1$	$1.00e^{-2}$	$3.66e^3$	$8.65e^2$
$H3,4$	$2.44e^5$	$1.53e^{-2}$	$2.29e^{-2}$	$2.10e^0$	$1.29e^2$	$1.88e^{-1}$	$1.57e^4$	$1.60e^{-1}$
$H3,5$	$2.44e^5$	$1.39e^{-2}$	$6.28e^{-2}$	$1.71e^0$	$1.04e^1$	$1.50e^{-2}$	$1.02e^1$	$6.73e^2$
parameter set	SSE	k_2	k_3	p_{-2}	p_{-3}	p_{-4}	p_{-5}	q_2
$H1,1$	$3.53e^5$	$4.71e^{-2}$	$6.82e^4$	$2.65e^1$	$3.21e^2$	$9.55e^{-2}$	$1.93e^2$	0
$H1,2$	$3.54e^5$	$5.16e^{-2}$	$1.17e^4$	$1.58e^0$	$2.76e^2$	$6.06e^2$	$7.50e^{-2}$	0
$H1,3$	$3.55e^5$	$4.79e^{-2}$	$1.62e^5$	$2.67e^1$	$5.32e^0$	$8.40e^2$	$6.85e^0$	0
$H1,4$	$3.55e^5$	$6.68e^{-2}$	$5.96e^3$	$1.03e^2$	$1.95e^{-1}$	$7.88e^2$	$5.63e^{-2}$	0
$H1,5$	$3.55e^5$	$5.23e^{-2}$	$7.88e^3$	$2.69e^0$	$4.19e^2$	$2.86e^2$	$8.07e^{-2}$	0
$H2,1$	$3.57e^5$	$4.94e^{-2}$	$3.00e^4$	$7.59e^1$	$3.06e^1$	$1.38e^1$	$1.12e^2$	$1.00e^0$
$H2,2$	$3.60e^5$	$4.94e^{-2}$	$3.42e^4$	$4.60e^1$	$8.77e^1$	$1.63e^2$	$1.03e^2$	$7.37e^3$
$H2,3$	$3.60e^5$	$5.05e^{-2}$	$1.75e^4$	$1.47e^2$	$4.12e^1$	$4.29e^2$	$2.37e^1$	$5.75e^3$
$H2,4$	$3.60e^5$	$5.16e^{-2}$	$5.74e^3$	$1.91e^2$	$3.97e^2$	$7.02e^1$	$5.87e^1$	$1.00e^0$
$H2,5$	$3.61e^5$	$5.30e^{-2}$	$7.58e^3$	$4.27e^1$	$4.84e^0$	$6.63e^2$	$2.15e^2$	$3.49e^0$
$H3,1$	$2.43e^5$	$6.08e^{-2}$	$7.11e^3$	$2.16e^1$	$9.81e^0$	$3.02e^{-1}$	$4.94e^{-2}$	$4.19e^2$
$H3,2$	$2.44e^5$	$6.59e^{-2}$	$3.41e^3$	$5.36e^1$	$4.52e^1$	$3.68e^{-2}$	$1.28e^1$	$5.83e^2$
$H3,3$	$2.44e^5$	$5.51e^{-2}$	$1.35e^4$	$1.07e^1$	$4.24e^2$	$3.78e^{-2}$	$1.00e^3$	$3.44e^2$
$H3,4$	$2.44e^5$	$6.02e^{-2}$	$5.82e^3$	$9.84e^0$	$3.20e^2$	$1.25e^0$	$8.72e^{-1}$	$5.22e^2$
$H3,5$	$2.44e^5$	$6.59e^{-2}$	$4.94e^3$	$8.34e^0$	$2.31e^{-1}$	$5.81e^2$	$5.02e^{-2}$	$6.29e^3$

Table J. Results of parameter fitting Model C to experimental observations of Syk Y525 and Y323 phosphorylation. Parameters sets H1,$1-5$ show the optimal results utilising the interactions described in hypothesis H1, Parameters sets H2,$1-5$ describe results utilising the interactions described in hypothesis H2 and parameter sets H3, $1-5$ utilising the interactions of H3. Results are from a sample of 1000 (rows $1-10$) and 10000 fits (rows $11-15$).

References

1. Best D, Senis YA, Jarvis GE, Eagleton HJ, Roberts DJ, Saito T et al. GPVI levels in platelets: relationship to platelet function at high shear. Blood. 2003;102(8):2811-18.

2. Burkhart JM, Vaudel M, Gambaryan S, Radau S, Walter U, Martens L, et al. The first comprehensive and quantitative analysis of human platelet protein composition allows the comparative analysis of structural and functional pathways. Blood. 2012;120(15):e73-e82.

3. Dimitrio L, Clairambault J, Natalini R. A spatial physiological model for p53 intracellular dynamics. Journal of theoretical biology. 2013;316:9-24.

4. Goldstein B, Faeder JR, Hlavacek WS, Blinov ML, Redondo A, Wofsy C. Modeling the early signaling events mediated by FcRI. Molecular Immunology. 2002;38(16):1213-19.

5. Faeder JR, Hlavacek WS, Reischl I, Blinov ML, Metzger H, Redondo A. et al. Investigation of early events in FcRI-mediated signaling using a detailed mathematical model. The Journal of Immunology, 2003;170(7):3769-81.

6. Huang L, Pan CQ, Li B, Tucker-Kellogg L, Tidor B, Chen Y et al. Simulating EGFR-ERK signaling control by scaffold proteins KSR and MP1 reveals differential ligand-sensitivity co-regulated by Cbl-CIN85 and endophilin. PloS one. 2011;6(8):e22933.

7. Kholodenko BN, Demin OV, Moehren G Hoek JB. Quantification of short term signaling by the epidermal growth factor receptor. Journal of Biological Chemistry. 1999;274(42):30169-181.

8. Kang M, Othmer HG. The variety of cytosolic calcium responses and possible roles of PLC and PKC. Physical biology. 2007;4(4):325.

9. Kim B, Hawes SL, Gillani F, Wallace LJ, Blackwell KT. Signaling pathways involved in striatal synaptic plasticity are sensitive to temporal pattern and exhibit spatial specificity. PLoS Comput. Biol. 2013;9(3):e1002953.

10. Miura Y, Takahashi T, Jung SM, Moroi M. Analysis of the interaction of platelet collagen receptor glycoprotein VI (GPVI) with collagen. J. Biol. Chem. 2002;277:46197204.

11. Owens ND, Timmis J, Greensted A, Tyrrell A. Elucidation of T cell signalling models. J Theor Biol. 2010;262(3):452-470.

12. Purvis, J. E., Chatterjee, M. S., Brass, L. F., & Diamond, S. L. (2008). A molecular signaling model of platelet phosphoinositide and calcium regulation during homeostasis and P2Y1 activation. Blood, 112(10), 4069-79.
